# Predicting Macrophage Spatial Localization from Single‐Cell Transcriptomes to Uncover Disease Mechanisms

**DOI:** 10.1002/advs.202410924

**Published:** 2026-02-28

**Authors:** Junping Yin, Qi Mei, Hans‐Joachim Paust, Ning Song, Yu Zhao, Daniela Klaus, Melanie Eichler, Yijun Hua, Jie Qin, Weiting Cheng, Christina K. Weisheit, Veronika Lukacs‐Kornek, Isis Ludwig‐Portugall, Sibylle von Vietinghoff, Christian F. Krebs, Johanna Klughammer, Ulf Panzer, Christian Kurts, Jian Li

**Affiliations:** ^1^ Institute of Molecular Medicine and Experimental Immunology (IMMEI) University Hospital Bonn 53127 Bonn Germany; ^2^ Department of Oncology Tongji Hospital Tongji Medical College Huazhong University of Science and Technology Wuhan Hubei 430030 P. R. China; ^3^ Cancer Center Shanxi Bethune Hospital Shanxi Academy of Medical Sciences Tongji Shanxi Hospital Third Hospital of Shanxi Medical University Taiyuan Shanxi 030006 P. R. China; ^4^ III. Department of Medicine University Medical Center Hamburg‐Eppendorf 20249 Hamburg Germany; ^5^ Renal Division Department of Medicine Beijing Anzhen Hospital Capital Medical University Beijing 100029 P. R. China; ^6^ Department of Nasopharyngeal Carcinoma Guangdong Key Laboratory of Nasopharyngeal Carcinoma Diagnosis and Therapy Sun Yat‐sen University Cancer Center Guangzhou 510060 P. R. China; ^7^ State Key Laboratory of Oncology in South China Sun Yat‐sen University Cancer Center Guangzhou 510060 P. R. China; ^8^ Department of Obstetrics Maternal and Child Health Hospital of Guangxi Zhuang Autonomous Region Nanning 530000 P. R. China; ^9^ Birth Defects Prevention and Control Institute Maternal and Child Health Hospital of Guangxi Zhuang Autonomous Region Nanning 530012 P. R. China; ^10^ Cancer Center Renmin Hospital of Wuhan University Wuhan Hubei 430060 P. R. China; ^11^ Department of Anesthesiology and Intensive Care Medicine University Hospital Bonn 53127 Bonn Germany; ^12^ Nephrology Section Medical Clinic 1 University Hospital Bonn 53127 Bonn Germany; ^13^ Hamburg Center for Translational Immunology University Medical Center Hamburg‐Eppendorf 20249 Hamburg Germany; ^14^ Gene Center and Department of Biochemistry Ludwig‐Maximilians‐Universität München 80539 Munich Germany; ^15^ Department of Microbiology and Immunology Doherty Institute for Infection and Immunity University of Melbourne Victoria 3000 Australia

**Keywords:** inflammation, kidney disease, macrophages, microglia, spatial immunology

## Abstract

When cells are isolated for single‐cell RNA sequencing (scRNA‐seq), their positional information is inevitably lost. Here, an algorithm termed MERLIN is described that can reconstruct such information in organs with compartmentalized anatomy, such as the kidney. Several independent immune cell scRNA‐seq datasets from three renal compartments were generated to train different machine learning algorithms. A modified multi‐layer Perceptron approach most accurately predicted positions of resident macrophages, best, achieving over 75% accuracy in both murine and human kidney datasets. More motile immune cells, like lymphocytes, were not predictable. Positional transcriptomic fingerprints were enriched in pathways of microenvironmental responses and cellular adaptation, and showed a sex bias. MERLIN also predicted positions of resident and recently recruited macrophages in a crescentic glomerulonephritis mouse model. Analysis of published scRNA‐seq datasets from endotoxin‐ and ischemia/reperfusion‐induced models of acute kidney injury revealed proinflammatory responses predominantly in outer medullary macrophages, consistent with the known pathology. Moreover, the response of cortical macrophages to commonly used therapies for diabetic nephropathy aligned with the known clinical drug efficacy. Finally, MERLIN was successfully trained to predict the spatial distribution of brain microglia. Together, MERLIN enables spatial interpretation of scRNA‐seq datasets in organs with defined anatomical regions and enhances mechanistic insights into disease processes.

## Graphical abstract

This study presents a machine‐learning framework entitled MERLIN that infers the microanatomical localization of macrophages from single‐cell transcriptomic data in structured organs such as the kidney and brain. Applying this method to existing datasets from models of acute kidney injury and diabetic nephropathy uncovered spatially resolved insights into disease pathophysiology.

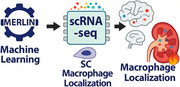

## 1 Introduction

Tissue‐resident immune cells perform sentinel functions against pathogens and support homeostasis in all organs.^[^ ^1^, ^2^ ^]^ Single‐cell RNA sequencing (scRNA‐seq) has provided new insights into their biology. However, during immune cell isolation for scRNA‐seq, the information regarding their cellular positioning within tissues is inevitably lost. That Information is critical for understanding macrophage functions, especially in organs like the kidney or brain, which are anatomically structured in distinct areas with unique functions.

The kidney can be visually distinguished into cortex, outer medulla (OM) and inner medulla (IM),^[^ ^3^, ^4^, ^5^ ^]^ which play distinct roles in water and electrolyte homeostasis.^[^ ^3^, ^5^ ^]^ The cortex contains numerous glomeruli that filter blood and channel the filtrate into a tubular network, where water, electrolytes and nutrients are reabsorbed.^[^ ^3^, ^6^ ^]^ Each of these compartments features a distinct microenvironment, shaped by increasing osmolarity and electrolyte concentrations and by decreasing oxygen and nutrient availability from cortex to IM.^[^ ^7^, ^8^, ^9^, ^10^ ^]^ Microenvironmental cues can shape the transcriptomes of intrinsic kidney cells and have been shown to imprint positional information, for example, in tubular epithelial cells.^[^ ^4^ ^]^ Such positional information, identified in highly variable expressed genes (HVG), has been used to infer the location of non‐immune kidney cells in renal compartments.^[^ ^4^ ^]^ It is generally assumed that the intrarenal position of immune cells cannot be predicted, because these are motile and may spend too little time in distinct areas to acquire microenvironmental cues.^[^ ^11^ ^]^ This particularly applies to immune cells recruited from the blood during inflammation or infection.

The kidney harbors an abundant network of resident immune cells, composed primarily of macrophages and dendritic cells (DCs), which populate the tubulointerstitial space and survey the microenvironment for signs of infection or injury.^[^ ^12^ ^]^ During kidney inflammation, monocytes infiltrate the kidney, differentiate to inflammatory macrophages and dendritic cells and modify local T cell responses. Glomerulonephritis initially targets the glomeruli, but inflammation later spreads over the entire kidney, and either heals or progresses and destroys the kidney. The most aggressive type, crescentic glomerulonephritis (cGN), has been studied extensively in animal models, revealing important roles for T cells, dendritic cells (DCs) and macrophages.^[^ ^11^ ^]^

Although spatial sequencing technologies are increasingly being applied to elucidate immune responses within intrarenal niches,^[^ ^13^, ^14^, ^15^, ^16^ ^]^ scRNA‐seq is still widely used due to its high resolution and cheaper price, despite the loss of positional information of cells.^[^ ^17^, ^18^, ^19^, ^20^ ^]^ Numerous scRNA‐seq datasets are deposited in public databases, whose interpretation would benefit from the ability to restore the lost positional information, even partially. However, tools that can extract such information from the transcriptomes of immune cells are missing.

Machine‐learning (ML) methods are widely used for artificial intelligence (AI)‐based prediction of big data.^[^ ^21^ ^]^ Several studies employed ML to improve the scRNA‐seq data analysis, for instance, cell clustering, multimodal integration and gene imputation.^[^ ^22^, ^23^, ^24^ ^]^ It was speculated that the use of neural networks in combination with attention mechanisms might render ML more effective and accurate in single‐cell data analysis.^[^ ^22^ ^]^ However, no study has yet employed ML to extract positional information from immune cells and predict their regional locations within organs based on scRNA‐seq. Here, we show that such data indeed contains spatial signatures that can be used to predict the regional location of macrophages in the kidney and the brain as examples for organs with compartmentalized anatomy.

## 2 Results

### 2.1 Generation of a Single‐Cell Resolution Immune Cell Dataset from the Kidney for Positional Analysis

**Figure 1:**
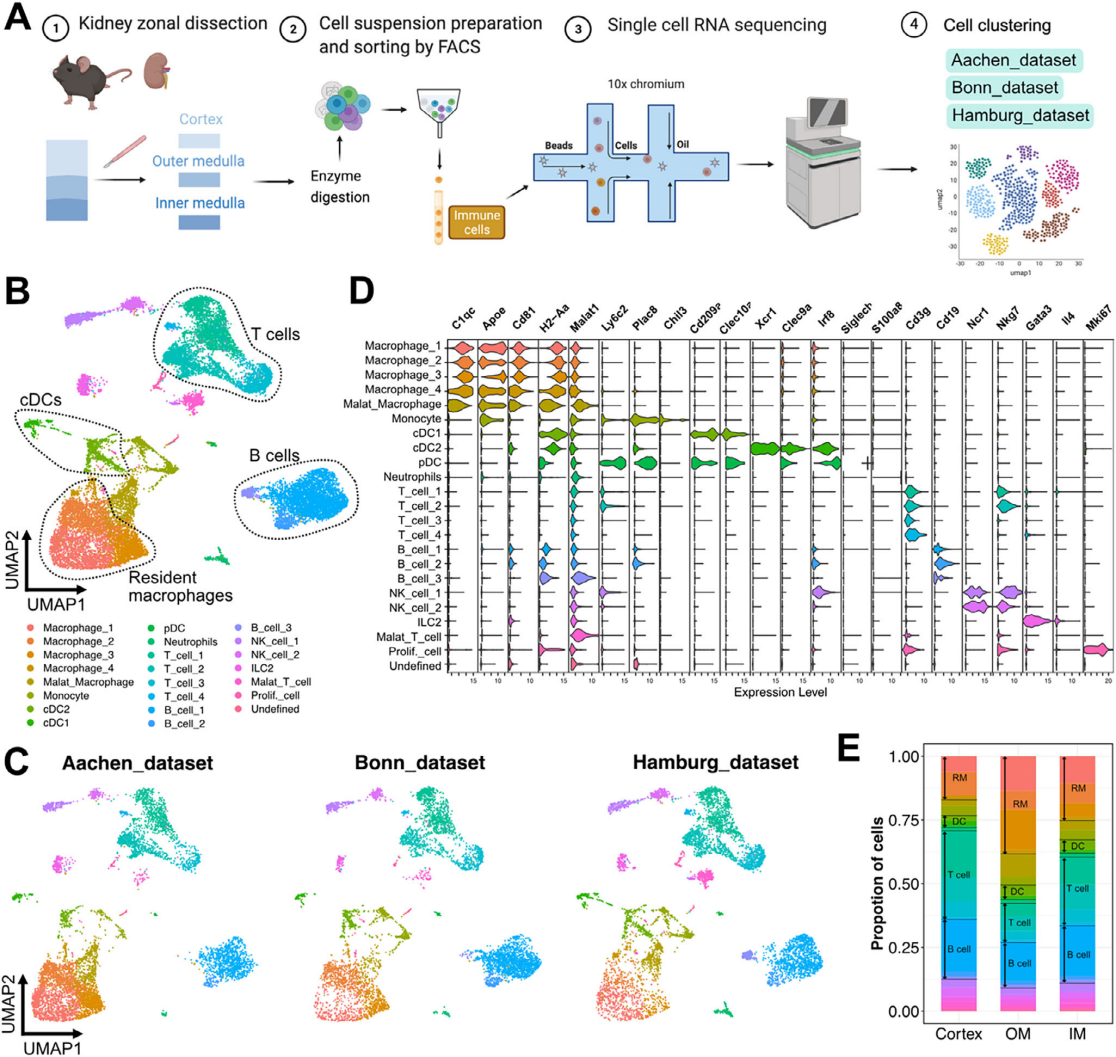
Positional scRNAseq of murine immune cells from the kidney. A) Schematic plot of positional scRNA‐seq of murine immune cells sorted from macro‐dissected compartments of kidney. B,C) Integrated (B) and split (C) UMAP plot of the Aachen, Bonn and Hamburg scRNA‐seq datasets showing the composition of immune cell clusters from healthy mouse kidneys. Resident macrophages, DCs, T cells and B cells were marked by dashed circles. D) Stacked violin plot showing the gene expression of single cell markers for annotation of immune cells. E) Cellular proportions of immune cells in cortex, OM (OM) and inner medulla (IM) of murine kidneys. Proportions of resident macrophage, DC, T cell and B cell were marked in the plot. RM, resident macrophage; DC, dendritic cell.

We manually dissected cortex, OM and IM, as three distinct regions from murine kidneys, prepared single cell suspensions and generated three (Aachen, Bonn and Hamburg) sequencing datasets using droplet‐based scRNA‐seq (**Figure** **1**A; Figure S1, and Table S1, Supporting Information). After filtering out cells with insufficient RNA quality, we obtained 20266 renal immune cells composed of 7147, 6158, and 6961 cells in Aachen, Bonn and Hamburg datasets, respectively (Figure 1B–D; Table S1, Supporting Information). Unsupervised clustering identified 23 clusters of immune cells (Figure 1B–D; Figure S2A,B, and Table S2, Supporting Information), which were categorized into two major types: mononuclear phagocytes consisting of resident macrophages (*C1qc*, *Cd81*, *H2‐Aa*),^[^ ^25^, ^26^, ^27^ ^]^ monocytes (*Ly6c2*, *Plac8*), DCs (*Cd209a*, *H2‐Aa, Clec10a*), a few neutrophils (*S100a8*) and lymphoid cells consisting of T cells (*Cd3g*), B cells (*Cd79a*), NK cells (*Ncr1*) and innate lymphoid cells (ILC2) (*Gata3*, *Il4*), respectively (Figure 1B). As expected, mononuclear phagocytes and lymphocytes were most abundant in all compartments. The frequency of resident macrophages was higher in the OM and IM compared to the cortex, while T cells were less frequent in OM and IM than in the cortex (Figure 1E; Figure S2C,D, Supporting Information).

### 2.2 ML‐Based Multinomial Classification of Positional Information from scRNAseq Data

**Figure 2:**
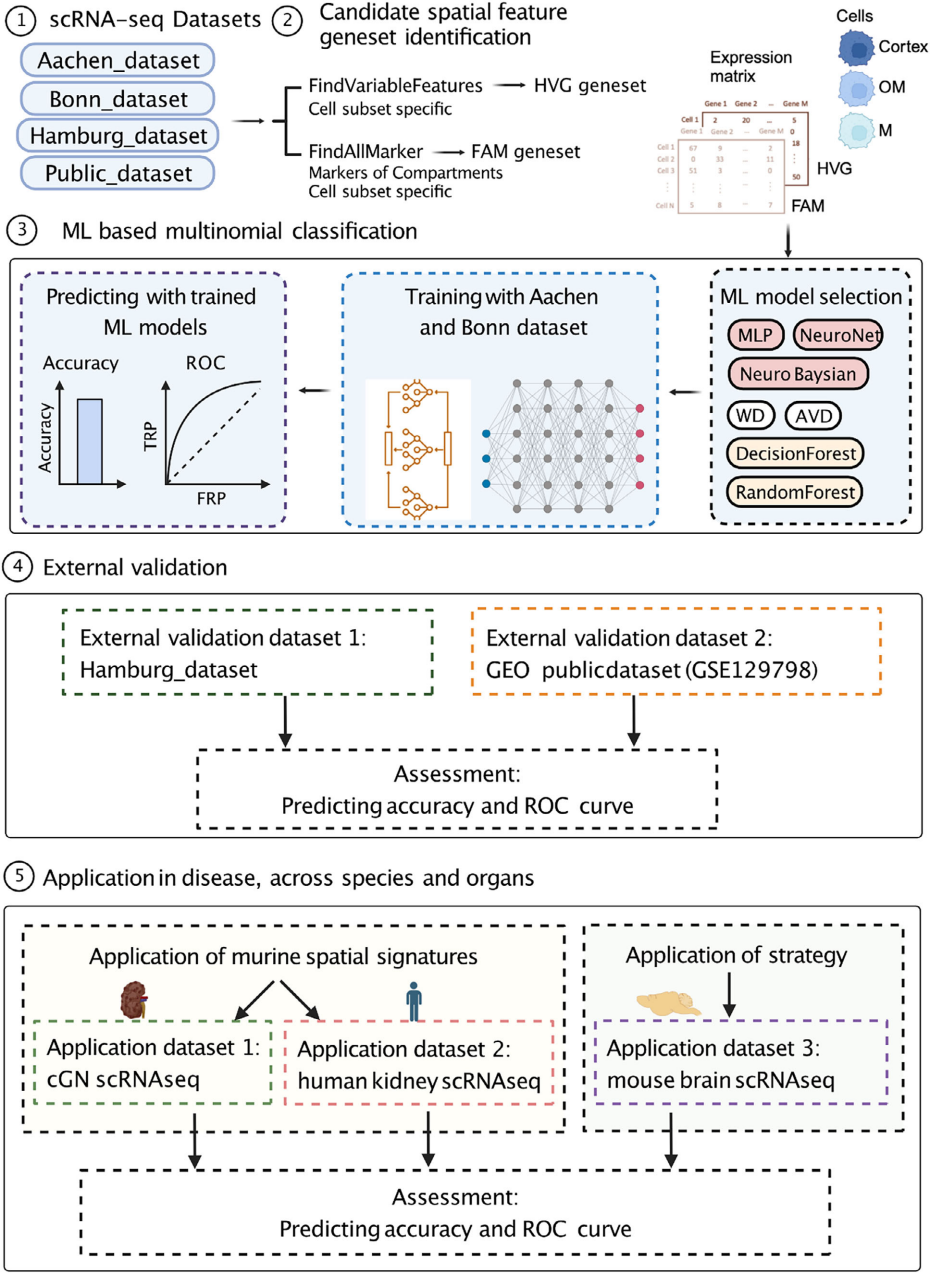
Schematic plot of the workflow for AI‐based multinomial classification of the positioning of renal immune cells and its application. Step 1: Generation of 3 new scRNA‐seq datasets and downloading of one public dataset from GEO database. Step 2: Extract potential positional signature genes by using two algorithms: FindVariableFeatures and FindAllMarker, which generated two genesets HVG geneset and FAM geneset. Expression matrices of HVG geneset and FAM geneset with positional annotation were extracted and used for downstream AI analysis. Step 3: To predict the positioning of renal immune cells, seven ML models were utilized to train the Aachen and Bonn datasets. Capability of models was assessed by overall accuracy and ROC curve for compartments. Step 4: External validation of prediction models by using the Hamburg and Public dataset. Step 5: To evaluate the application of positional signatures in kidney disease and kidneys from other species, and application of the strategy for identifying positional signatures of other organs, we produced a scRNA‐seq dataset from kidneys of mice with experimental cGN and downloaded a scRNA‐seq dataset for human kidneys and scRNA‐seq dataset for the brain.

**Figure 3:**
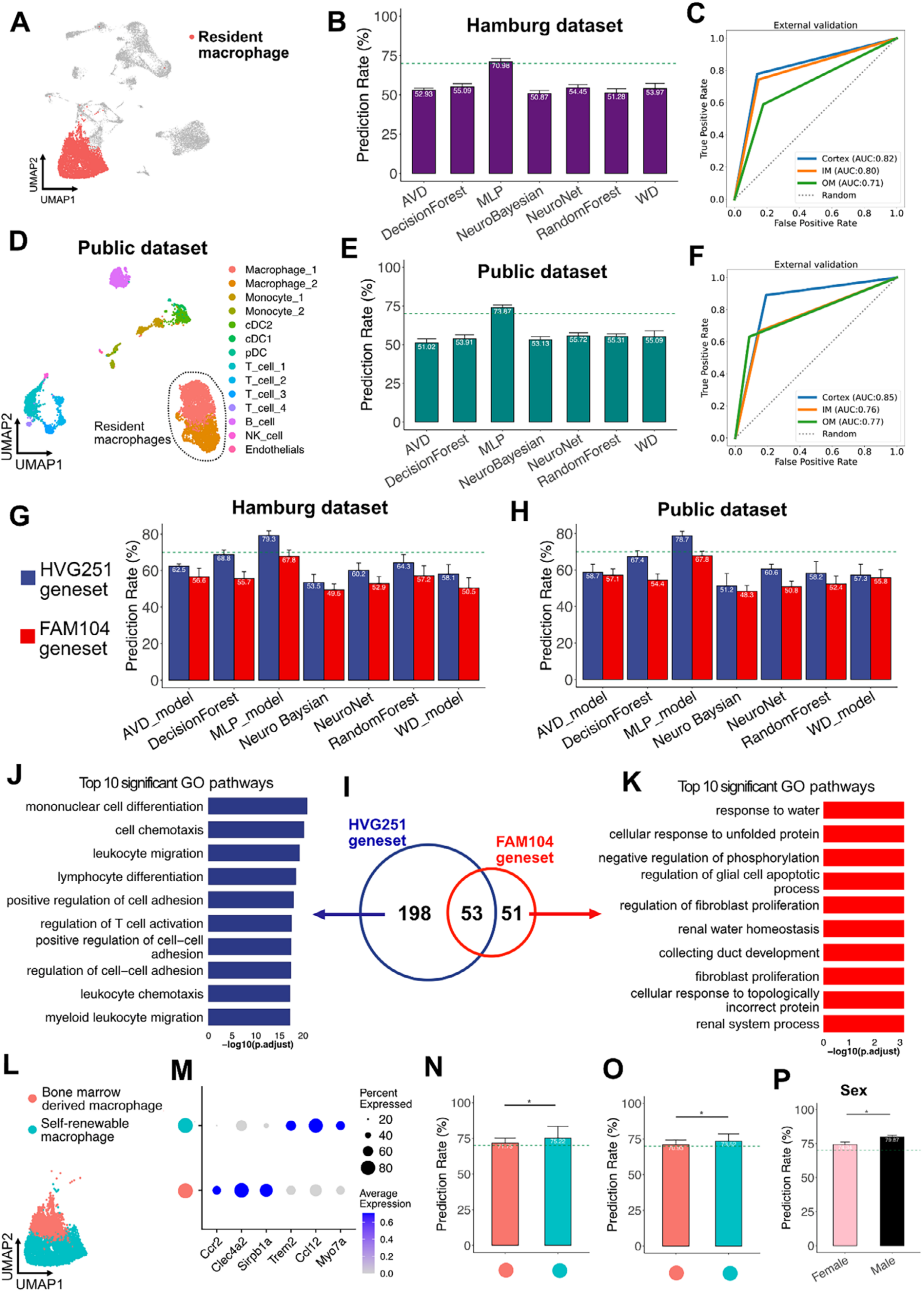
Predicting the regional location of kidney immune cells from external datasets. A) UMAP plot highlighting resident macrophages in the kidney. B) Predicting the accuracy of the HVG137 geneset in the external Hamburg dataset by using ML models trained on resident macrophages from the 2 training datasets. The Hamburg dataset contributed to HVG137 selection; therefore, it was excluded from model training and used solely for validation. C) ROC curve of MLP model on predicting the location of resident macrophages from the Hamburg dataset. AUC: area under curve. (D) UMAP plot of the Public dataset of renal immune cells from healthy mice downloaded from a public database. Resident macrophages were marked by dashed circle. E) Predicting accuracy of the HVG137 geneset in the external Public dataset by using ML models trained on resident macrophages from the 2 training datasets. F) ROC curve of MLP model on predicting the location of resident macrophages from Public dataset. AUC: area under curve. G,H) Predicting accuracy of HVG251 (blue) and FAM104 (red) geneset in external Hamburg dataset (G) or Public dataset (H) by using ML models trained on resident macrophages from the 2 training datasets. I) Venn diagram of HVG251 (blue) and FAM104 (red) geneset showing the shared and distinctive genes of each geneset. Numbers of genes in each group were labelled. J,K) Top10 of significantly enriched GO pathways of genes of HVG251 (J) and FAM104 geneset (K). L,M) UMAP plot (L) and marker gene expression (M), of bone marrow‐derived or self‐renewable resident macrophages in the Hamburg dataset. N,O). Predicting accuracy of HVG251 for bone marrow‐derived or self‐renewable resident macrophages in the Hamburg dataset. (N) and from Public dataset (O). P) Predicting the accuracy of HVG251 for resident macrophages from male and female mice in the public dataset. Data presented as Median ± SEM. Statistical significance was determined by using Student's *t*‐test. P value, *, 0.05.

HVG of kidney tubular epithelial cells have recently been shown to contain positional information.^[^ ^4^ ^]^ Therefore, we identified and merged the top100 HVG genes from the training datasets and generated the first candidate geneset HVG137 (**Figure** **2**; Table S3, Supporting Information). Renal resident macrophages are relatively immotile and long‐lived cells,^[^ ^11^, ^28^, ^29^ ^]^ suggesting that their transcriptome might more likely acquire positional information from the microenvironment. Therefore, we first investigated the positional signatures of such macrophages (**Figure** **3**A). Seven ML models were trained on the expression matrix HVG137 from training datasets, and all of them were able to predict the positions of resident macrophages with an accuracy over 70%, supporting the validity of the ML model‐based approach (Figure S3, Supporting Information).

Next, we validated the predictive capacity of these trained ML models in two external datasets. When testing with the use of the Hamburg dataset, the MLP model trained on the HVG137 geneset predicted with a median accuracy of 70.98%, whereas the other models showed less than 70% (Figure 3B). The ROC curve showed that the AUC values of MLP model were above 0.7 in all three compartments (Figure 3C).

To further validate this model, we analyzed an additional public dataset,^[^ ^30^ ^]^ which profiled 6437 immune cells containing 14 clusters, including resident macrophages, DC, T cells, B cells, etc (Figure 3D; Figure S4A,B, Supporting Information). Consistent with the findings above, resident macrophages were more frequent in the medulla compared to the cortex, while T cells are more abundant (Figure S4C,D, Supporting Information). When we applied the trained ML models to the public dataset, the MLP model of HVG137 achieved 73.87% of accuracy, and AUC values were also above 0.7 in all three compartments (Figure 3E,F). Additionally, we tested other contemporary machine learning models, XGBoost, support vector machines (SVM). XGBoost achieved comparable accuracy (70.12%) to our MLP model, while SVM showed slightly lower performance (65.32%), likely due to the high dimensionality and non‐linear structure of the data. In summary, these findings indicated that the HVG genes of resident macrophages carry positional information.

To extract as many genes carrying such positional information as possible, we merged the top100 HVG genes in all training and predicting datasets and generated a new HVG geneset with potentially improved predicting capability, termed HVG251 (Table S3, Supporting Information). We trained the HVG251 geneset with the two independent datasets and validated them with the two external datasets. As expected, the HVG251 geneset achieved higher predicting accuracy of 79.3% and 78.7% in the Hamburg and public datasets, respectively (Figure 3G,H). The AUC values of the MLP model of HVG251 were above 0.75 in all compartments of both datasets (Figure S5A,B, and Table S4, Supporting Information). Using a randomized gene set for the same prediction task resulted in consistently poor predictive performance (Figure S5C,D, Supporting Information). Two additional feature selection methods, recursive feature elimination (RFE) and mutual information‐based ranking (MIR) were deployed to identify two gene sets, RFE251 and MIR251, respectively. Using both gene sets for the same prediction tasks resulted in a comparable but slightly weaker predictive performance (Figure S5E–H, Supporting Information). These comparison results indicated implied that HVG251 achieves a significantly improved predicting accuracy. The HVG251‐based algorithm, integrated with MLP model, was designated as MERLIN (Machine Learning‐Enabled Regional localization of Leukocytes) and subsequently applied for downstream analysis.

To determine whether we can extract genes carrying positional information with other approaches, we identified genes differentially expressed among the three compartments by using the *FindAllMarker* (FAM) and obtained geneset FAM104 (Figure 2; Table S3, Supporting Information). We trained the FAM104 geneset using the training datasets and predicted using the Hamburg dataset and Public dataset. However, this showed poor predicting capability with less than 70% accuracy in all ML models (Figure 3G,H), suggesting that HVG was superior to other gene sets in identifying positional signatures.

### 2.3 Analysis of the Genes Used by MERLIN and the Influence of Ontology and Sex

We next wished to know details about the geneset HVG251 and FAM104. We observed that only 53 genes overlapping between them, which accounted for 21% of HVG251 and 51% of FAM104 (Figure 3I; Table S5, Supporting Information). Gene ontology (GO) enrichment analysis showed that the shared genes in both datasets were significantly enriched in regulation of TNF production and monocyte chemotaxis, in the HVG251 geneset only in GO pathways of mononuclear cell differentiation, cell chemotaxis and leukocyte migration, while the non‐overlapped genes of FAM104 geneset were significantly enriched in responses to water, to unfolded protein response and regulation of phosphorylation (Figure 3J,K; Figure S6, and Tables S6–S8, Supporting Information). Additionally, significantly enriched GO terms with HVG251 included responses to hypoxia, oxidative stress and regulation of ROS production (Table S9, Supporting Information), all of which are closely associated with the hypoxic microenvironment of the kidney.^[^ ^31^ ^]^

It has been reported that renal resident macrophages are derived from either bone marrow or precursors from the yolk sac.^[^ ^32^ ^]^ Yolk sac‐derived resident macrophages self‐renew in the kidney and possess a transcriptome distinct from that of bone marrow‐derived macrophages.^[^ ^29^, ^33^, ^34^ ^]^ By utilizing the markers described in a recent study,^[^ ^34^ ^]^ we were able to discriminate resident macrophages into yolk sac derived macrophages (self‐renewable macrophages) and bone marrow‐derived macrophages (Figure 3L,M; Figure S7A–D, Supporting Information). The accuracy for predicting the position of self‐renewing macrophages was higher than that of bone marrow‐derived macrophages, in both external validation datasets (Figure 3N,O).

We noted that the Public dataset contained cells from male and female mice with similar proportions of resident macrophages (Figure S7E,F, Supporting Information). It has been reported that sex can affect the transcriptomes of renal epithelial cells,^[^ ^30^ ^]^ suggesting that the HVG251 geneset derived from male mice might possess a distinct capability to predicting the positioning of cells from female mice. Indeed, the accuracy and AUC of ROC curves for all three compartments for predicting the positioning of male resident macrophages were significantly superior than in female mice, indicating that sex needs to be considered when positional prediction datasets are defined (Figure 3P).

### 2.4 MERLIN Predicts Macrophages Positions also Within the Human Kidney

**Figure 4:**
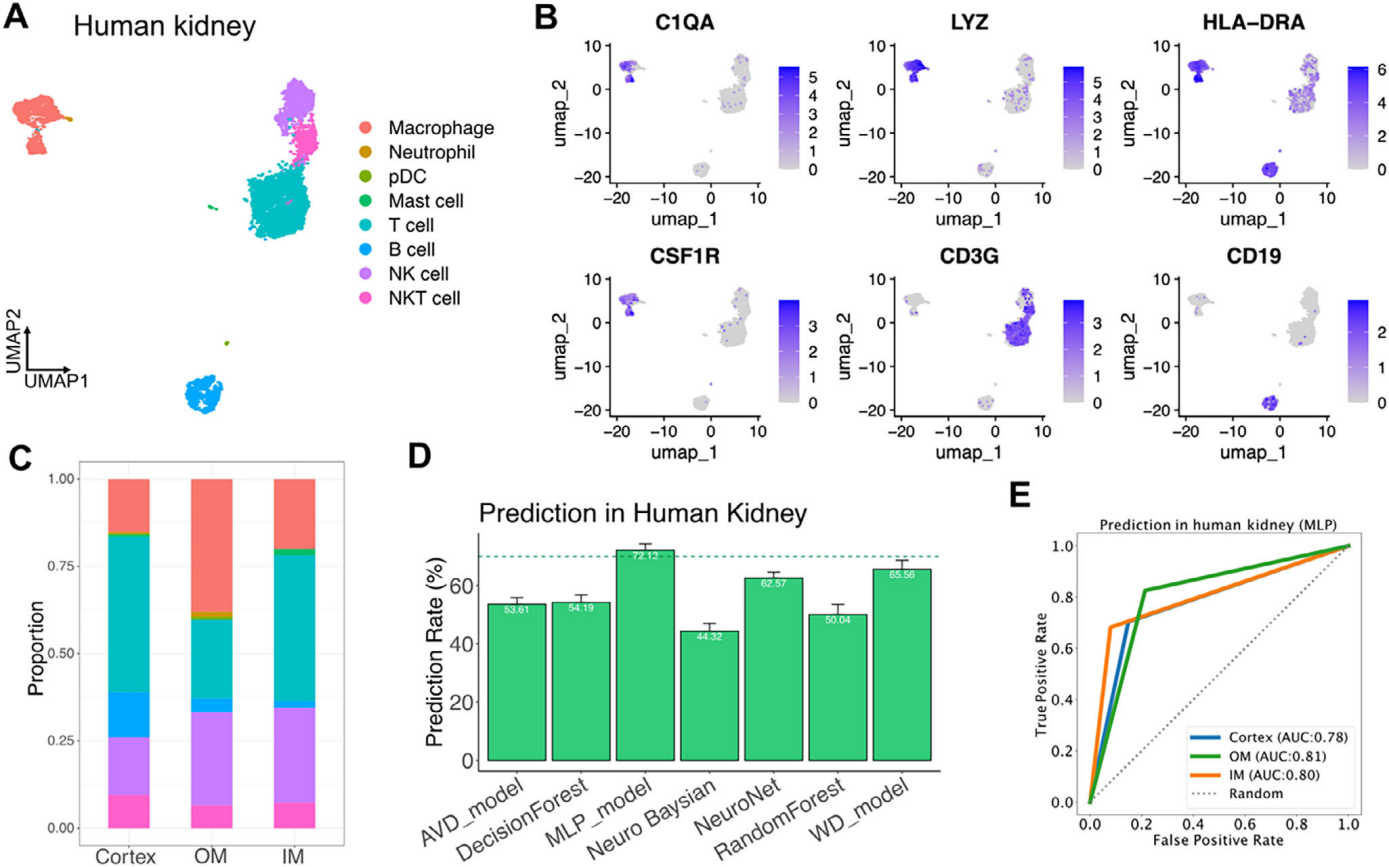
MERLIN predicts macrophages positions also within the human kidney. A) UMAP plot of scRNA‐seq dataset of immune cells from human kidneys. Resident macrophages were marked by dashed circle. B) Featureplot showing the gene expression of single cell markers for annotation of cell subsets of human renal immune cells. Light gray indicated low expression, and blue high expression. C) Proportions of renal immune cells in cortex, OM and IM of human kidney. D) Predicting the accuracy of the mouse‐human common HVG210 geneset for resident macrophages in the human kidney dataset. (E) ROC curve of MLP model on predicting the location of resident macrophages from the human kidney dataset. AUC: area under the curve.

Since the transcriptome of resident macrophages in the kidney has been reported to be conserved across species,^[^ ^35^ ^]^ we aimed to assess the applicability of MERLIN for analyzing human scRNA‐seq datasets. To this end, we downloaded and analyzed a human scRNA‐seq dataset with positional information of renal immune cells.^[^ ^5^ ^]^ This dataset profiled 7803 immune cells including macrophages, neutrophils, as well as T cells and B cells (**Figure** **4**A,B). Consistent with the findings in mouse kidneys, more macrophages were present in the medulla compared to the cortex, and more T cells in the cortex (Figure 4C).

Before applying the ML models of HVG251 trained with murine data to the human kidney dataset, we utilized the homoloGene database to align genes between both species and generated a mouse‐human common HVG210, after discarding 41 mouse genes that have no human homology. Using the HVG210, we applied the ML models and found that the MLP model predicted the spatial positions of human renal macrophages with an accuracy of 72.12% (Figure 4D), and the AUC values exceed 0.78 across all renal compartments (Figure 4E), indicating that the conserved mouse–human HVG210 gene set possesses robust predictive capability for human kidney macrophages, comparable to its performance in the murine dataset.

### 2.5 MERLIN Predicts Microglia Positions in the Brain

**Figure 5:**
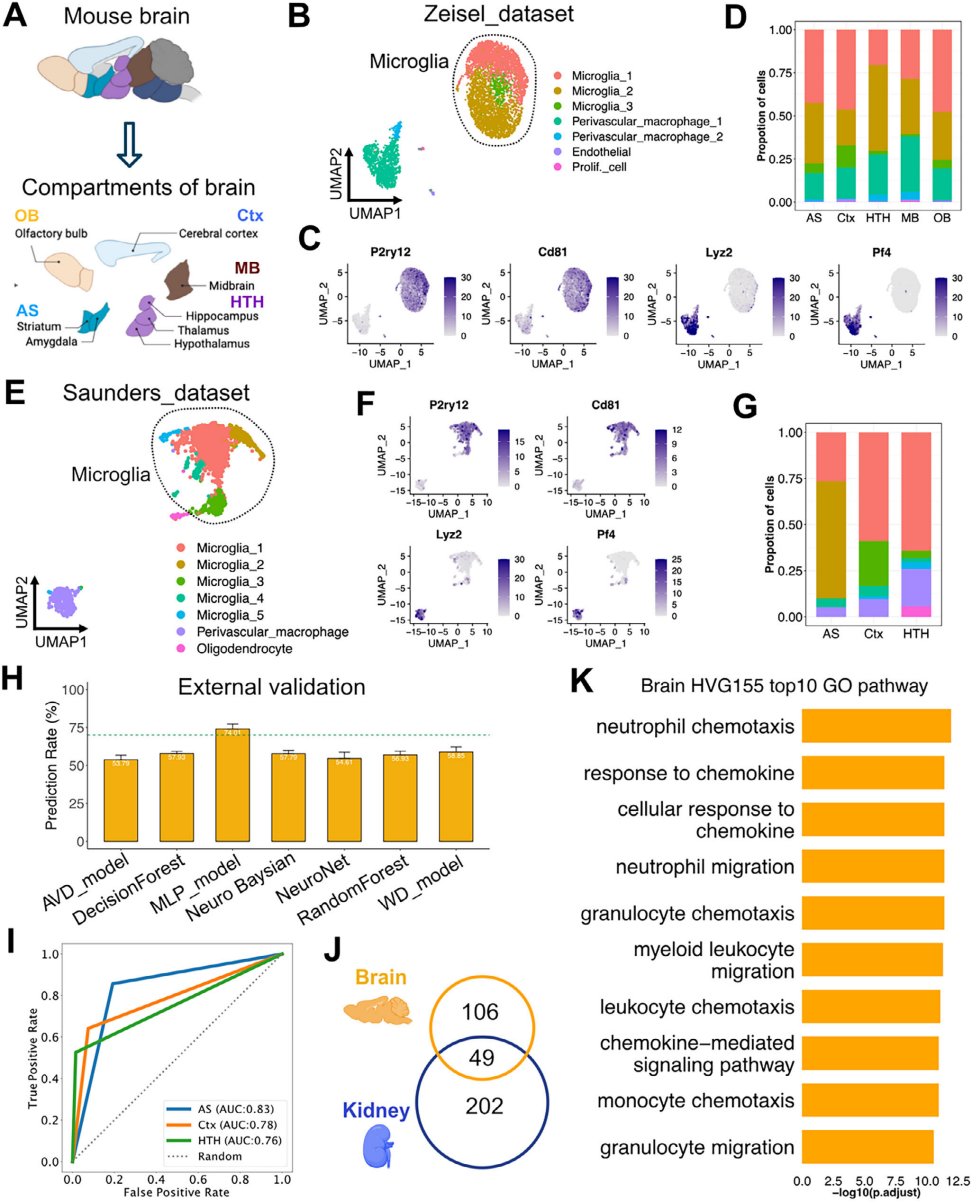
MERLIN predicts microglia positions in the brain. A) Schematic plot of dissected compartments of the brain: Olfactory bulb (OB), cerebral cortex (Ctx), midbrain (MD), amygdala‐striatum (AS), hippocampus‐thalamus‐hypothalamus (HTH). B) UMAP plot of scRNA‐seq Zeisel_dataset of brain immune cells. C) Featureplots showing gene expression of single cell markers for annotation of cell subsets of human brain immune cells in Zeisel_dataset. Light gray indicated low expression while navy blue indicated high expression. D) Cellular proportion of immune cells in each brain compartment Zeisel_dataset. E) UMAP plot of scRNA‐seq Saunders dataset of brain immune cells. F) Featureplots showing the gene expression of single cell markers for annotation of cell subsets of human brain immune cells in Saunders_dataset. Light gray indicated low expression while navy blue indicated high expression. G) Cellular proportion of immune cells in each brain compartments in Saunders dataset. H) Predicting accuracy of external validation of brain HVG155 on microglia in Saunders dataset. I) ROC curve of MLP model on predicting the location of microglia from murine Saunders dataset. AUC: area under curve. J) Venn diagram of murine brain HVG155 (orange) and kidney HVG251 (blue) geneset showing the shared and distinctive genes of each geneset. Number of genes in each group were labelled. K) Top10 significantly enriched GO pathways of genes in brain HVG155 geneset.

To assess the broader utility of MERLIN, we extended our analysis to the brain, an organ characterized by highly specialized regional architecture that may influence immune cell distribution.^[^ ^36^ ^]^ Its resident immune cells are mainly microglia cells, which possess a remarkable histological resemblance to kidney‐resident macrophages. Depending on their positioning, they may play diverse roles in various neurological disorders.^[^ ^37^ ^]^ Recently, Zeisel et al published a scRNA‐seq dataset of cells from brain compartments (**Figure** **5**A).^[^ ^36^ ^]^ That dataset contained 5081 immune cells from 5 regions of the brain, including olfactory bulb, cerebral cortex, midbrain, amygdala‐striatus, hippocampus‐thalamus‐hypothalamus, and consisted mostly of microglia (Figure 5B–D). Another dataset from Saunders et al.^[^ ^38^ ^]^ contained 2125 immune cells from three brain regions: cerebral cortex, amygdala‐striatum and hippocampus‐thalamus‐hypothalamus (Figure 5E–G). As in the kidney, we determined the HVG genes from both datasets and merged the top 100 HVGs from each. This resulted in a combined set of 155 HVGs specific to brain microglia (Table S11, Supporting Information).

To independently validate the predicting capability of the 155 brain HVGs, we trained the ML models of HVGs on the Zeisel_dataset and predicted in the Saunders_dataset. Using this HVGs, the MLP model achieved a prediction accuracy of 74.01% (Figure 5H), with the AUC values above 0.76 across all brain compartments in the Saunders_dataset (Figure 5I). This highlights the capacity of the HVG signature to capture the spatial identity of macrophages across distinct organs, extending its predictive utility beyond the kidney to the brain.

Furthermore, although only 49 genes overlapped between the kidney and brain HVG sets (Figure 5J), the brain microglia HVGs were also significantly enriched for biological processes such as cell chemotaxis and myeloid cell migration—mirroring the enrichment observed in kidney HVGs (Figure 5K; Table S12, Supporting Information). These findings demonstrated that MERLIN can capture spatially informative transcriptional programs across another organ with compartmentalized architecture.

### 2.6 MERLIN Predicts Macrophage Positions in Experimental Glomerulonephritis

**Figure 6:**
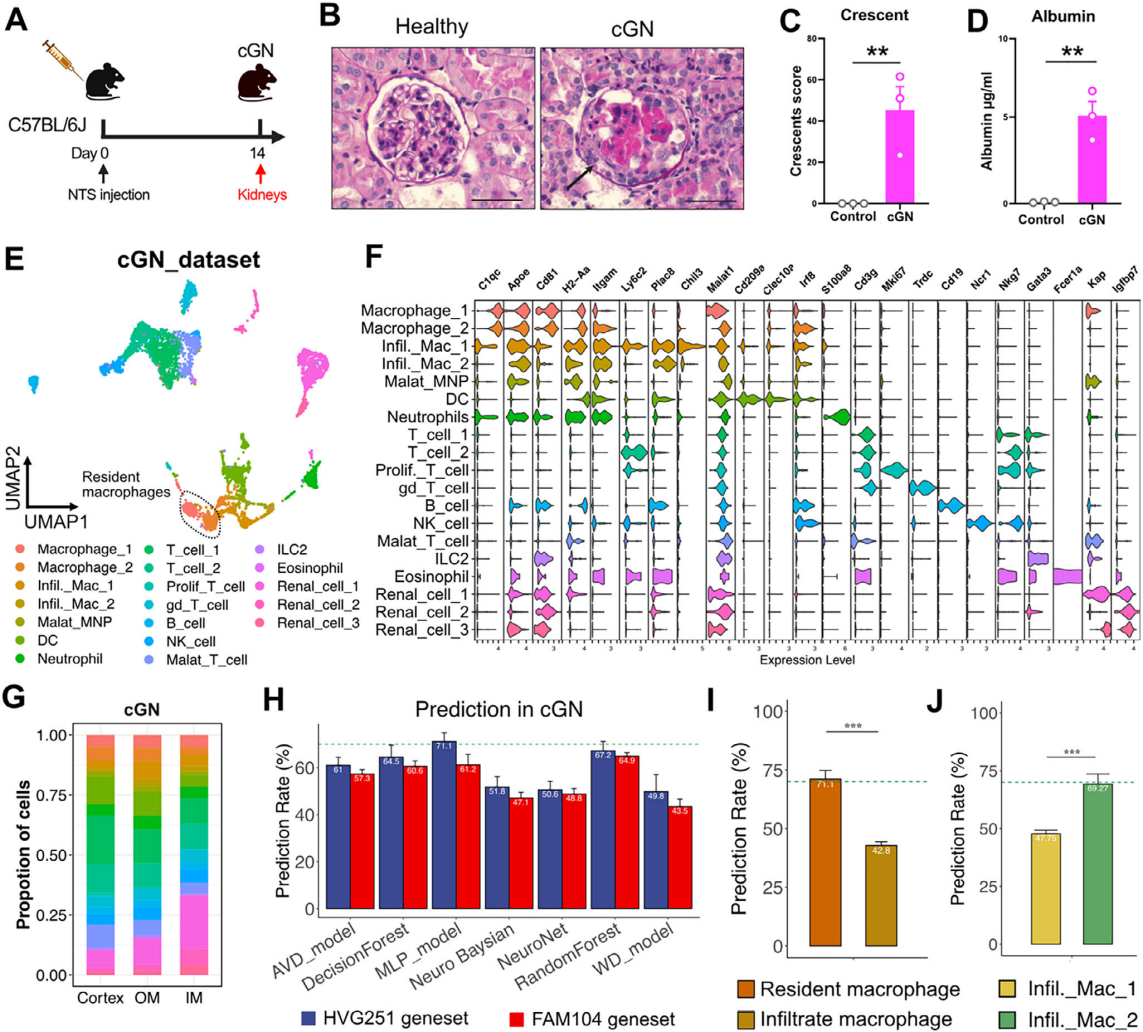
MERLIN predicts macrophage positions in experimental glomerulonephritis. A) Experimental plan for cGN in the C57BL/6J mice. Kidneys of mice were analyzed at day 14 after induction. B) Histology picture of PAS‐stained kidney sections from healthy and cGN mice. Arrow indicating a crescent in the glomerulus. Scale bar, 50um. C,D) Quantitative analysis of kidney crescents score of kidneys (B) and urine albumin concentration (C) from healthy and cGN mice. E) UMAP plot of scRNA‐seq dataset of renal immune cells from experimental cGN mice at day 14 after inducing experimental cGN. F) Stacked Violin plot showing the gene expression of single cell markers for annotation of cell subsets of renal immune cells. G) Cellular composition of cell subsets of renal immune cells in cortex, OM and IM of kidney from cGN mice. H) Predicting accuracy of HVG251 (blue) and FAM104 (red) geneset of resident macrophages in cGN dataset. I) Predicting accuracy of HVG251 geneset of monocyte‐derived infiltrating and resident macrophages in the cGN dataset. J) Prediction accuracy based on HVGs of recently and previously infiltrated macrophages.

Next, we investigated whether MERLIN is also applicable to inflammatory kidney disease, given that the phenotype, proliferative capacity, and functional state of resident macrophages are potentially modulated by inflammatory microenvironments.^[^ ^29^, ^39^, ^40^ ^]^ It is unknown whether such inflammatory cues may alter positional signatures of resident macrophages. To study this, we induced cGN in mice (**Figure** **6**A), which causes the formation of glomerular crescents and proteinuria (Figure 6B–D), typical for this disease.^[^ ^12^ ^]^ We profiled 5402 single cells from day 14 of cGN (Figure 6E,F; Figure S8A,B, and Table S10, Supporting Information). Consistent with previous studies,^[^ ^11^, ^41^ ^]^ the majority of inflammatory immune cells in experimental cGN were resident macrophages, DCs, monocyte‐derived infiltrating macrophages, T cells and B cells (Figure 6G). Compared to healthy mice, resident and infiltrating macrophages as well as DCs became more abundant in the cortex (Figure S8C, Supporting Information), which is expected since glomeruli are located in the cortex.

To test whether the positional signatures from healthy mice can predict locations of resident macrophages during kidney disease, we applied the ML models described above to cGN datasets. We observed a slight reduction of the predicting accuracy of the MLP model of HVG251 to 71.14% (Figure 6H), and AUC values of each compartment were still above 0.75 in the cGN dataset (Figure S9, Supporting Information), indicating that acute inflammation affects positional fingerprints of resident macrophages in cGN, but this influence was limited. These findings suggest that the positional signatures of kidney‐resident macrophages were conserved in cGN.

Monocyte‐derived macrophages infiltrate the kidney in experimental cGN and differentiate to macrophages.^[^ ^11^, ^42^ ^]^ While doing so, they might acquire positional signatures. However, when we predicted the positions of monocyte‐derived infiltrating macrophages with the MLP model trained with the HVG251 geneset from resident macrophages from healthy mice, accuracy was low (Figure 6I). We hypothesized that infiltrating macrophages might acquire positional signatures distinct from those of resident macrophages, and determined their specific HVG geneset in cGN (Table S3, Supporting Information) for training the MLP machine learning model. Only 17 genes overlapped between the HVG genesets from resident and infiltrating macrophages (Table S3, Supporting Information). We next hypothesized that monocyte‐derived infiltrating macrophages might require time to acquire distinct positional signatures during cGN. Therefore, we distinguished two subsets of infiltrating macrophages in our scRNA‐seq dataset, the subset infil. Mac_1 with higher expression of monocyte features like *Ly6c*, *Plac8* and *Chil3*,^[^ ^43^ ^]^ and the subset infil._Mac_2 with lower expression of these features (Figure 6F), assuming that macrophages that had the time to downregulate monocyte features^[^ ^44^ ^]^ might acquire a positional fingerprint during that time. Indeed, this approach predicted the locations of infil._Mac_2, but not of infil._Mac_1, approaching 70% in cGN (Figure 6J). These findings indicated that infiltrating macrophages acquire a cell type‐specific positional signature and that such acquisition requires some time.

### 2.7 Prediction of Dendritic Cells and Lymphocyte Positions in Kidney Inflammation

We wished to determine whether our approach can also predict the location of other immune cells in the kidney, such as conventional DCs (cDCs), another type of mononuclear phagocytes.^[^ ^11^ ^]^ When we trained the ML models using cDCs’ HVG on the training dataset, none of them predicted the positioning of cDCs in the Hamburg or the Public dataset (Figure S10A,B, Supporting Information), implying the HVG genes of cDCs did not carry enough extractable positional information.

A recent study suggested that cDCs in many tissues can be classified as a new subset termed cDC3, which arises from monocyte‐DC progenitors, in contrast to cDC2 that originate from common DC progenitors.^[^ ^45^ ^]^ We noted that a large number of renal cDCs indeed expressed marker genes of such cDC3, like *Fcgr3*, *Sirpa* and *Tmem172a*, while only few expressed the cDC2 marker *Cd7* (Figure S10C, Supporting Information), suggesting that many renal cDCs comply with the classification as cDC3. As shown above, the position of such cells is less predictable than that of tissue‐resident cells, likely because they are constantly recruited, resulting in a high turnover that leaves little time to acquire positional fingerprints. Furthermore, numbers of renal DCs are much lower than those of macrophages, reducing statistical prediction power.

Moreover, HVGs from T cells and B cells did not carry positional fingerprints, as low accuracy was observed when the trained ML models of HVGs from T cells or B cells were applied to predict the positioning of T cells or B cells in the external dataset (Figure S10D–G, Supporting Information). ILCs are thought to be tissue‐resident, but are too rare in the healthy kidney to train the ML algorithm. Likely, motile renal leukocytes do not spend enough time in a kidney compartment to acquire positional information.

### 2.8 Predicting Macrophage Positions from Published scRNA‐seq Datasets Provides Mechanistic Insights into Acute Kidney Injury

**Figure 7:**
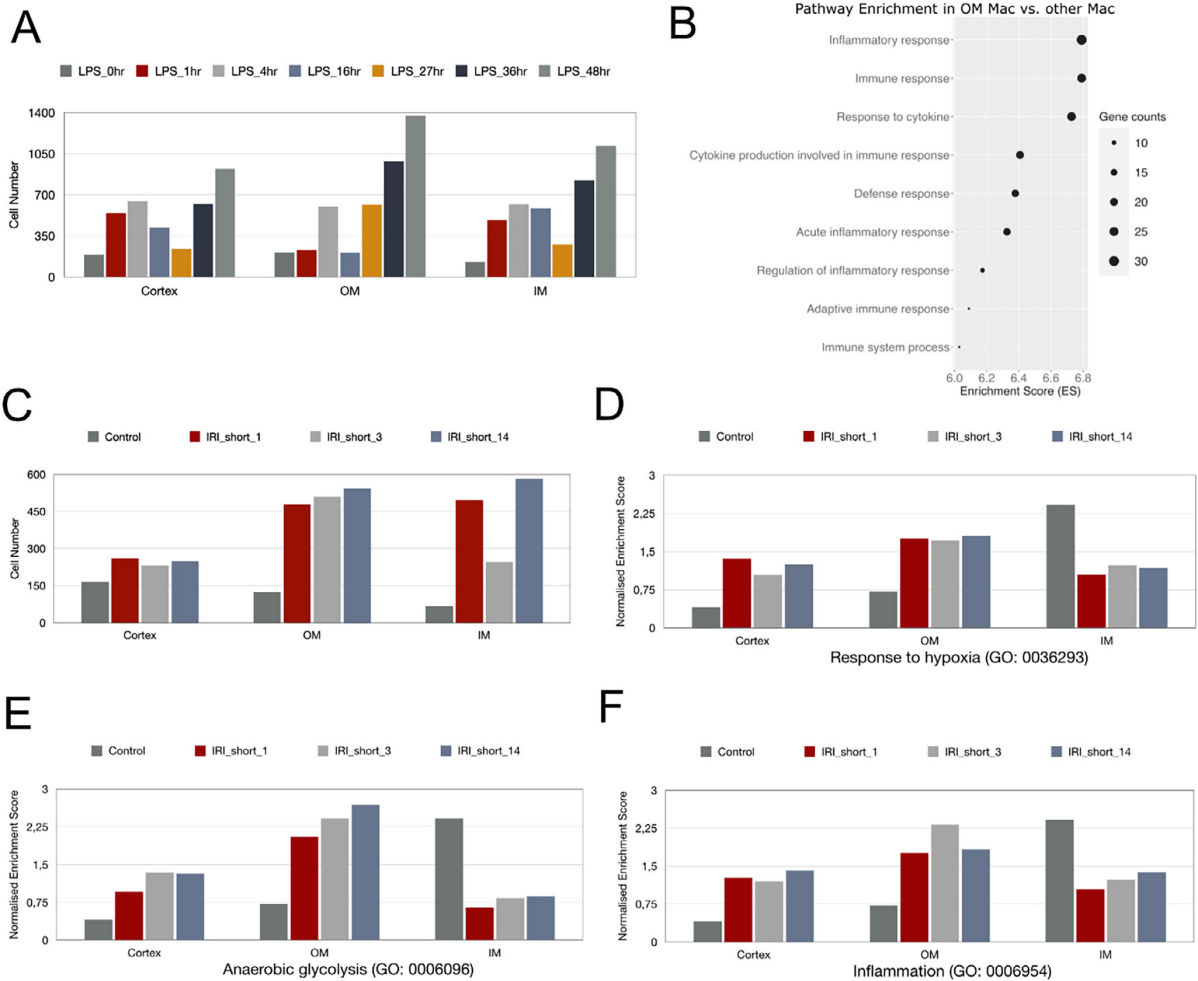
Predicting macrophage positions from published scRNA‐seq datasets provides mechanistic insights into acute kidney injury. A) Time kinetics of normalized cell counts in the cortex, OM and IM of the kidney before and after LPS. B) Gene enrichment analysis showing enriched pathways of differentially expressed genes between macrophages in OM and other compartments after LPS. C,D) Bar plots showing normalized cell counts (C), enrichment scores of hypoxia pathways (D), anaerobic glycolysis E), and inflammatory pathways F) in the cortex, OM and IM of kidneys from control and IRI mice.

Next, we wished to use MERLIN to gain mechanistic insights into other kidney diseases by analyzing published scRNA‐seq datasets. A previous study presented a transcriptomic atlas of immune cells in acute septic kidney failure.^[^ ^46^ ^]^ Across cortex, OM and IM, macrophage counts were predicted to change markedly over the 48‐h LPS timeline with differing kinetics. In all regions, numbers deviated from baseline within the first hours, followed by transient troughs at ∼16 h and ∼27 h, and a subsequent rebound by 36–48 h (**Figure** **7**A). These dynamics align with the organ‐wide, phase‐structured response in septic shock, where early inflammatory signaling is followed by a period of network “shutdown” and reduced cell–cell communication ≈16–27 h, before recovery programs emerge.^[^ ^46^ ^]^ Previous immunohistochemistry studies had reported that the OM is most, and the cortex is least affected in endotoxin‐induced kidney injury.^[^ ^47^, ^48^, ^49^ ^]^ Consistently, analysis with MERLIN showed minimal macrophage accumulation in the cortex and pronounced infiltration in the OM (Figure 7A). The genes involved in inflammatory, immune and cytokine responses were more highly expressed in OM macrophages compared to those from other renal regions (Figure 7B).

Another publication contained transcriptomes of immune cells at 1, 3 and 14 days after ischemia/reperfusion injury (IRI).^[^ ^50^ ^]^ Analysis with MERLIN revealed that macrophage numbers increased rapidly after short duration IRI across all regions, with the strongest expansion in the OM, followed by IM and cortex (Figure 7C). Interestingly, macrophage numbers in short IRI declined in the IM after 3 days, but increased again after 14 days (Figure 7C). IM macrophages expressed genes involved in the hypoxia response already before IRI, whereas an upregulation was seen in all compartments after inducing IRI (Figure 7D). Macrophages in the OM, and lesser so, in the cortex showed the highest enrichment for anaerobic glycolysis and inflammatory response signatures (Figure 7E), consistent with their reported ability to use this pathway to generate energy under low oxygen conditions.^[^ ^51^ ^]^ This ability may push macrophages into glycolysis‐driven expression of proinflammatory genes (Figure 7F), known to promote kidney injury in IRI,^[^ ^39^, ^52^ ^]^ explaining why IRI‐induced damage is observed primarily in the cortex and OM. Together, these findings validated MERLIN in 2 more kidney disease models and allowed new mechanistic insights.

### 2.9 MERLIN Elucidates Therapeutic Responses in Experimental Diabetic Nephropathy

**Figure 8:**
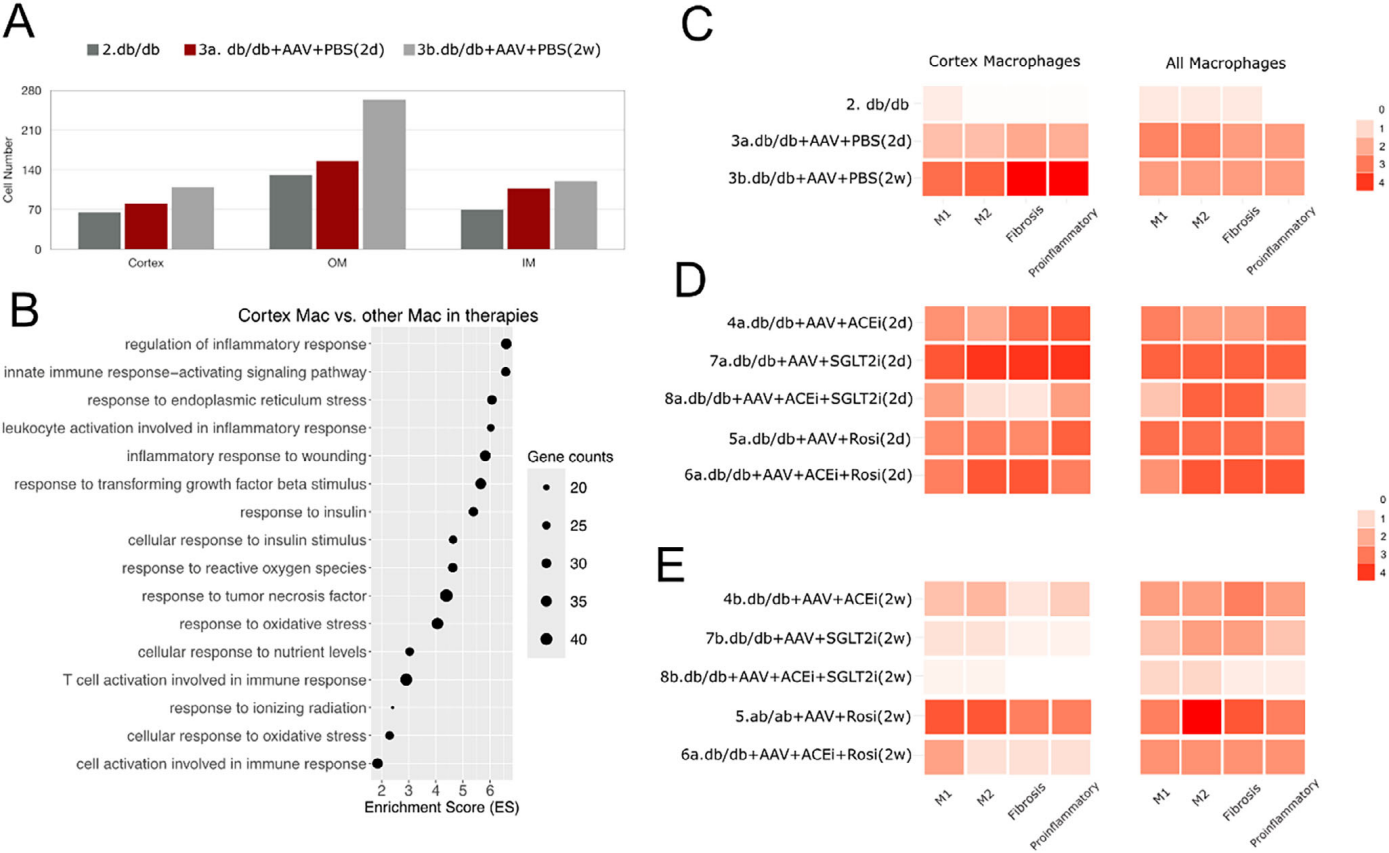
MERLIN elucidates therapeutic responses in experimental diabetic nephropathy. A) Time kinetics of normalized cell macrophage in the cortex, OM and IM of the kidney in db/db mice 2 days or 2 weeks after injection of an AAV expressing renin. B) Gene enrichment analysis showing enriched pathways of differentially expressed genes between macrophages in the Cortex and other compartments. C–E) This panels display heatmaps illustrating the scores of M1‐like and M2‐like macrophage polarization, fibrotic activity, and proinflammatory signaling in macrophages without (C) and with treatment 2 days (D) or 2 weeks (E) after injecting AAV.

Finally, we used MERLIN to analyze an accelerated diabetic nephropathy (DN) model, incorporating scRNA‐seq data 2 days or 2 weeks following disease initiation and therapeutic intervention.^[^ ^53^ ^]^ MERLIN predicted that macrophages accumulated in all compartments, especially in the OM (**Figure** **8**A), consistent with previous studies implicating medullary macrophages in DN progression.^[^ ^54^, ^55^ ^]^

A clinical hallmark manifestation of DN is glomerulosclerosis, i.e. a process occurring in the cortex. We therefore focused our analysis on cortical macrophages and found a disproportional activity in pathways involved in M1 and M2 differentiation, in fibrosis and inflammation (Figure 8B), all of which have been implicated in DN pathogenesis.^[^ ^54^, ^56^ ^]^ This was confirmed by pathway enrichment analysis, which showed in cortical macrophages high enrichment of several inflammatory, stress and fibrosis responses (Figure 8B), all of which have been implicated in DN pathogenesis.^[^ ^54^, ^56^ ^]^

The dataset also included groups of mice treated with an ACE inhibitor, an SGLT2 inhibitor, a glitazone, and various combinations. All treatments led to a reduction in macrophage numbers across renal compartments, with the most pronounced effect observed under combination therapy (Figure S11, Supporting Information). When we examined pathogenic programs, MERLIN revealed a consistent upregulation across all pathogenic programs in cortical macrophages at two days after disease induction in cortical macrophages (Figure 8C). This upregulation was strongest with the SGLT2 inhibitor, intermediate with ACE inhibition, and mild with rosiglitazone. Notably, the ACE+SGLT2 combination produced an even more pronounced upregulation than either monotherapy. In contrast, whole‐kidney macrophage analysis showed only muted or absent changes, substantially underestimating the early response (Figure 8D). These observations align with clinical phenomena, including the transient “eGFR dip” observed after initiating SGLT2 inhibitors due to adenosine‐mediated afferent vasoconstriction,^[^ ^57^, ^58^ ^]^ and the modest creatinine rise after ACE inhibition, reflecting efferent arteriolar dilation.^[^ ^59^, ^60^ ^]^ Rosiglitazone treatment is less effective in patients, consistent with the minor early changes observed here.^[^ ^61^, ^62^ ^]^

By two weeks, cortical macrophages displayed attenuation of the same programs across all treatment groups (Figure 8E). The SGLT2 inhibitor induced the strongest suppression of proinflammatory and fibrotic signatures, ACE inhibition showed a delayed but clear reduction, and rosiglitazone produced only modest improvements without full normalization (Figure 8E). The combination of ACE and SGLT2 inhibition achieved the most profound effect, nearly normalizing all four GO terms. In whole‐kidney analyses, these week‐2 improvements appeared blunted and inconsistent, masking the magnitude and timing of treatment‐specific responses (Figure 8E). These analyses illustrated that the analysis of cortical macrophages in DN more closely mirrors clinical observations than whole‐kidney macrophage analysis.^[^ ^61^, ^62^ ^]^

## 3 Discussion and Conclusion

The present study demonstrates that the transcriptomes of kidney‐resident immune cells carry spatial information that allows predicting their regional location.^[^ ^4^, ^5^ ^]^ To show this, we profiled the transcriptomes of 20266 immune cells from several independent scRNA‐seq datasets isolated from three anatomical compartments of healthy murine kidneys. By employing ML models, we extracted positional information, which allowed predicting qualitatively the position of tissue‐resident and late infiltrating macrophages with good accuracy depending on the ML algorithm used.

For feature selection as input for ML models, we applied both *FindAllMarkers* and *FindVariablesFeatures* methods from the Seurat package and the HVG251 generated by *FindVariableFeatures* contains more positional information than FAM104 produced by *FindAllMarkers*. A possible explanation could be that the FAM104 set includes only the most deregulated genes, consistent with recent studies.^[^ ^4^ ^]^ Pathway enrichment analysis indicated that HVG251 was enriched in pathways associated with leukocyte migration and chemotaxis, which is plausible given that these mechanisms guide the migration and positioning of immune cells.^[^ ^63^, ^64^, ^65^ ^]^ We also examined transcription factors and metabolic genes within HVG251 that may mediate compartment‐specific macrophage behavior. For example, Hif1a, Nrf2, and Slc2a1 were more highly expressed in medullary macrophages, consistent with adaptation to hypoxic and glycolytic environments. The selection of highly variable genes (HVGs) was central to MERLIN's predictive framework. While HVGs are traditionally used for cell‐type discrimination in scRNA‐seq, our findings suggest that they also encode positional information in long‐lived, tissue‐resident immune cells. Comparative analyses showed that HVG‐based models outperformed those using random genes, DEGs (FAM104), RFE251, and MIR251, supporting their relevance for spatial inference.

Among the seven ML models tested, MLP proved most effective in extracting the positional fingerprint. MLP is based on a neural‐artificial network.^[^ ^66^ ^]^ Our study explored another utilization of this model by modifying the attention mechanism for dealing with scRNA‐seq data, as proposed by a previous study.^[^ ^22^ ^]^ The attention mechanism maximizes the learning efficacy while minimizing information loss at each step during training. This may explain why the modified MLP performed better than the other ML algorithms examined here. The suitability of a ML model depends on various factors, including quality and quantity of data, feature selection, hyperparameter tuning, ensemble method, evaluation metrics and others. Importantly, it was necessary to combine two independent scRNA‐seq datasets to avoid inherent overfitting phenomena. We validated our approach using seven widely used ML models, performed internal 100 folder cross‐validation and used two additional external validation datasets. It is possible that training with more than 2 scRNA‐seq datasets can further improve performance.

A recently published tool, NovoSpaRc, has been reported to extract positional information from scRNA‐seq data from tissue cells.^[^ ^4^, ^67^ ^]^ However, we found that this tool failed to predict the locations of renal macrophages (data not shown). This may be explained by the design of NovoSpaRc, which is specialized at reconstructing the location of non‐hematopoietic immotile cells. However, macrophages are motile, albeit less so than lymphocytes for instance. Thus, MERLIN was unable to predict the positions of T and B cells, which may be explained by the limited time these motile cells have for acquiring a positional fingerprint typical for a given microenvironment. Unexpectedly, MERLIN also failed to extract positional fingerprints from cDCs, although these cells are tissue‐resident to some extent. This may be due to the finding that renal cDCs arise from circulating monocyte‐progenitors, as postulated in a recent study, which designated such cells as DC3.^[^ ^45^ ^]^ In addition, DCs are not long‐time resident as macrophages are, but enter, exit and move through the kidney, limiting their time to integrate positional information into their transcriptomes.^[^ ^11^, ^28^ ^]^ Finally, the number of DCs in the kidney is much lower than that of macrophages, which reduces statistical power. In our benchmarking, we used recommended pipelines and default parameters for NovoSpaRc, but did not perform extensive hyperparameter tuning or retraining. As such, the comparison should be interpreted as a qualitative reference rather than a definitive performance ranking. Future work may involve adapting these frameworks specifically for immune cell localization and conducting more rigorous head‐to‐head evaluations under matched conditions. Currently, existing mapping algorithms such as Tangram,^[^ ^68^ ^]^ CellTrek,^[^ ^69^ ^]^ and CytoSPACE^[^ ^70^ ^]^ align scRNA‐seq data with spatial transcriptomic data from the same source to improve tissue cartography and sequencing resolution. By contrast, MERLIN differs in both purpose and implementation. It uses previously generated scRNA‐seq training data with anatomical annotation to subsequently infer positional information from independent scRNA‐seq datasets. Once such training datasets are available, Merlin only needs scRNA‐seq data, and no spatial transcriptomic scaffolds from the same source.

When we analyzed immune cells in a glomerulonephritis model, MERLIN still predicted resident macrophages positions, albeit with slightly reduced accuracy. It also permitted predicting the positions of infiltrating macrophages that had spent some time within the kidney to acquire positional transcriptome information. Notably, sex disparity was evident as well, implying that the sex of the training and prediction datasets needs to be considered.

At this point, we used the MERLIN analytic pipeline to analyse several published scRNAseq datasets from kidney disease models such as IRI or endotoxin shock.^[^ ^71^ ^]^ MERLIN accurately predicted the OM as the compartment in which macrophages are most activated and where histopathological damage is most severe. The biphasic macrophage trajectory in endotoxin shock maps onto the sepsis timeline, where the kidney transitions from inflammatory activation to temporary communication collapse and then to repair.^[^ ^46^ ^]^ The relatively low increase in the cortex aligns with a recent study concluding that macrophage–endothelial crosstalk in the cortex limits local inflammatory damage during sepsis,^[^ ^49^ ^]^ further supporting region‐specific behavior.

Analysis of the IRI dataset revealed a regionalized macrophage response dominated by accumulation and glycolytic/inflammatory programs in the OM. These findings align with the unique oxygen biology of the OM, where the corticomedullary gradients and countercurrent exchange render the thick ascending limb and adjacent interstitium containing macrophages chronically hypoxia‐prone and sensitive to IRI.^[^ ^72^ ^]^ Hypoxia signaling and glycolytic activation in OM macrophages suggest HIF‐driven immunometabolic reprogramming toward a pro‐inflammatory phenotype, which is well known to promote pathology in IRI.^[^ ^73^, ^74^, ^75^ ^]^ Interestingly, IM macrophages showed strong hypoxia signaling before IRI, consistent with the extreme dysoxia in the IM. Only their numbers declined after IRI, suggesting that hypoxia‐ or nutrient‐stress–induced death as a plausible cause, given that renal macrophage pyroptosis has been documented in IRI, and that its inhibition is renoprotective.^[^ ^76^ ^]^ Future studies examining region‐resolved macrophage death pathways in IRI may test this hypothesis.

Analysis of a scRNAseq dataset of diabetic mice receiving different treatments further illustrated how compartmentalized analysis with MERLIN can facilitate mechanistic discoveries. We found that analysis of cortical macrophages provided a sharper and more clinically relevant window into drug responses during DN than whole‐kidney macrophage analyses. All therapies triggered early proinflammatory and fibrotic programs 2 days after treatments, most striking with SGLT2 inhibition and most modest with rosiglitazone. Combination therapy produced an amplification not captured by total macrophage analysis. These dynamics reflect known early hemodynamic perturbations in patients: SGLT2 inhibitors cause a transient eGFR dip due to restoration of tubuloglomerular feedback;^[^ ^57^, ^58^, ^77^ ^]^ ACE inhibitors induce a modest early creatinine rise via efferent vasodilation.^[^ ^59^, ^60^ ^]^ Rosiglitazone, by contrast, exerted weaker and slower effects explained by systemic insulin sensitization and fluid‐retention.^[^ ^61^, ^62^ ^]^ By two weeks, cortical macrophages showed attenuation of inflammatory and fibrotic programs with all therapies, strongest under SGLT2 inhibition, delayed with ACE inhibition and modest with rosiglitazone. Combination therapy nearly normalized these programs. These transcriptional dynamics parallel clinical outcomes, where SGLT2 inhibitors and ACE inhibitors are known to slow DN progression, while thiazolidinediones provide only limited renal benefit.^[^ ^60^, ^77^, ^78^ ^]^ Thus, cortical macrophage profiling with MERLIN captured biphasic treatment responses involving acute aggravation followed by attenuation and the differential clinical effectivity of the three drug classes studied,^[^ ^61^, ^62^ ^]^ which more closely mirrored clinical observations than whole‐kidney macrophage analysis. As with any statistical analysis, these findings do not establish causality between cortical macrophage activation and clinical drug responses in DN. Nevertheless, the correlation was remarkable, and causality should be tested in future studies.

MERLIN also allowed regional analysis in the brain, another organ with distinct macro‐anatomical structure,^[^ ^79^, ^80^ ^]^ extending the application scope of MERLIN to other organs. Interestingly, the HVG within the transcripts of murine kidney and brain were both highly enriched in pathways related to mononuclear phagocyte differentiation, leukocyte migration and chemotaxis. This suggests that the positional information‐carrying genes may be similar across organs.

Studying further organs will require the generation of scRNA‐seq training datasets. Doing so by dissecting macro‐anatomically discernable compartments, as done here, allows only microanatomical positional prediction, which is much inferior to “true” spatial sequencing technologies. However, it may be possible to increase the resolution by generating training datasets using such spatial technologies. Thereby, MERLIN can complement true spatial techniques by its ability to utilize the numerous scRNA‐seq datasets from mice and humans with various diseases published in online repositories. Furthermore, MERLIN can process entire organs, whereas spatial sequencing techniques can only examine a small area of an organ in a tissue section and may miss areas of pathological importance, unless numerous sections are analyzed, which is time‐ and very cost‐intensive.

In conclusion, we present MERLIN as a robust, cost‐efficient, and effective method for inferring the regional localization of macrophages from their single‐cell RNA transcriptomes in both physiological and pathological contexts. Our findings demonstrate that regional analysis of macrophage transcriptomes can yield novel pathophysiological insights and therapeutic targets. Although the present study could only begin to address the complexities of the kidney diseases studied here, the results are consistent with current knowledge and extend them by new, testable hypotheses for future investigation. MERLIN may provide a useful framework for elucidating the roles of resident immune cells in the kidney, the brain, and, prospectively, in other organ systems. Validation in organs with more complex anatomy or in disease microenvironments will be important future steps. Furthermore, extending MERLIN to additional datasets, including multiplexed imaging, may allow extending its application across diverse tissue architectures. While MERLIN is not currently positioned as a clinical diagnostic tool, it may offer scientific usefulness by enabling spatially resolved analysis of immune cell states from scRNA‐seq data. In disease models such as glomerulonephritis, ischemia/reperfusion injury, and diabetic nephropathy, MERLIN revealed compartment‐specific macrophage programs, including hypoxia signaling, glycolytic activation, and fibrotic responses, that are not apparent in whole‐kidney analyses. These findings provided mechanistic insight into regional immune dynamics and may inform therapeutic targeting. As spatial transcriptomics and single‐cell technologies become more integrated into translational research, tools like MERLIN can help bridge molecular profiling with anatomical context, supporting the development of precision medicine strategies that account for tissue microenvironment and immune cell localization.

## 4 Experimental Section

### Mice

Male 10‐week‐old C57BL/6J mice were purchased from Charles River and housed in the animal facility of the RWTH Aachen University, University Clinic Bonn and University Clinic Hamburg‐Eppendorf. Nephrotoxic nephritis was used as an experimental cGN mouse model and induced by treating the mice with nephrotoxic serum derived from sheep immunized with mouse kidney cortex extracts as previously described.^[^ ^41^, ^81^ ^]^ To collect the urine, mice were put individually in metabolic cages overnight. The level of urine albumin was determined using ELISA kit (Biomol, E99‐134). Animal experiments were approved by the Landesamt für Natur, Umwelt und Verbraucherschutz of North Rhine‐Westphalia, Germany.

### Single Cell RNA Sequencing of Renal Immune Cells

Murine kidneys from healthy or cGN mice were perfused with ice‐cold PBS, dissected into cortex, OM, and IM, and digested in RPMI 1640 medium with collagenase and DNase. The resulting single‐cell suspension was filtered, stained with LIVE/DEAD™ dye and CD45 antibody, and sorted for immune cells using BD ARIA III. Sorted cells were loaded into a 10x Genomics Chromium chip, libraries were prepared using the 10× Genomics Single‐Cell Gene Expression Kit, and sequenced on Illumina NovaSeq 6000 after quality control.

### Analysis of Single‐Cell RNA Sequencing Data

The raw sequencing reads were demultiplexed using 10× Genomics Cell Ranger, generating FASTQ files. Alignment and quantification followed, producing a gene expression matrix mapped to the mm10 reference genome. The matrix was then imported into Seurat (v4.0, R v4.1) for processing, integrating datasets from healthy and cGN mice using IntegrateData. Cells were filtered based on gene count (200–4000) and mitochondrial content (>20% removed; Figure 1). LogNormalization, scaling, PCA (2000 variable genes, vst method), and clustering were performed with Seurat's defaults. UMAP (20 PCs, resolution 0.5) visualized clusters, which were annotated using SingleR (ImmGen) and known biological markers as recently described.^[^ ^81^ ^]^ The annotated gene expression matrix was extracted for machine learning analysis.

### Generation of Highly Variable Expressed Genes (HVGs)

HVGs were calculated specifically for resident macrophages within each training dataset (Aachen and Bonn) using the FindVariableFeatures function in Seurat v4.0, with default parameters (mean.var.plot method, selection of top 100 genes per dataset). These HVGs were then merged to generate the HVG137 and HVG251 gene sets.

### External Validation of Murine Renal Immune Cells

For external validation, we used the Hamburg dataset and a public single‐cell RNA‐seq dataset (GSE129798) from the GEO database. The Public dataset included murine immune cells from kidney compartments of healthy C57BL/6J mice, sequenced using 10× Genomics Chromium v2. Both datasets were processed using Seurat in R, following the original authors’ settings, and their expression profiles were extracted for multinomial classification validation with machine learning models.

### Application of Spatial Signatures in Human Kidney

To measure the predictive accuracy of murine spatial signatures across species, we downloaded a scRNA‐seq dataset of mature human kidney with compartment information from a published paper. In this dataset, the human kidneys were dissected into cortex, OM and IM as we did on the murine kidneys. Single cells from these three compartments were prepared by enzymatic digestion and profiled by using Chromium single cell 3’ kit. Gene expression profiles of a certain immune cells subset were extracted and utilized for prediction of positioning of renal immune cells.

### Statistics

All statistics were performed in the R software (Version 4.1). Student's *t*‐test was applied to determine the statistical significance of the difference between two individual groups. Multiple testing correction was done by using Benjamini‐Hochberg (BH) adjustment. A *p*‐value lower than 0.05 was considered as statistically significant.

### Data Availability

The scRNA‐seq datasets generated in this study have been deposited in the Gene Expression Omnibus (GEO) database of the National Center for Biotechnology Information and are accessible via accession number GSE262968. The published scRNA‐seq datasets of murine kidney, human kidney and murine brain used in this study are accessible in the original studies Endotoxin injury GSE151658;^[^ ^46^ ^]^ IRI GSE180420;^[^ ^50^ ^]^ diabetic nephropathy GSE184652.^[^ ^53^ ^]^ Further data generated in the present study are available in the Supplementary material. The source code is available in the figshare database with the following link: https://doi.org/10.6084/m9.figshare.28599722.

## Conflict of Interest

The authors declare no conflict of interest.

## Author Contributions

J.Y. and Q.M. are first authors. C.K., and J.L. are joint senior authors. J.Y., J.L. and C.K. conceptualized the study and wrote the paper. J.Y., H.J.P., N.S., S.L., D.K., and M.E. performed the experiments. J.Y., Y.Z., W.C. and J.L. performed the bioinformatic analysis. I.L‐P., S.v.V, J.K., C.F.K., J.Q., Y.H., C.W., V.L.K. and U.P. provided essential tools and knowhow. Q.M., C.F.K., U.P., C.K. and J.L. acquired funding. All authors discussed and interpreted the data.

## Supporting information



Supporting Information

Supplemental Table 1

Supplemental Table 2

Supplemental Table 3

Supplemental Table 4

Supplemental Table 5

Supplemental Table 6

Supplemental Table 7

Supplemental Table 8

Supplemental Table 9

Supplemental Table 10

Supplemental Table 11

## Data Availability

The scRNA‐seq datasets generated in this study have been deposited in the Gene Expression Omnibus (GEO) database of National Center for Biotechnology Information and are accessible via accession number GSE262968. The published scRNA‐seq datasets of murine kidney, human kidney and murine brain used in this study are accessible in the original study. The other data generated here are available in the Supplementary material. The source code is available in the figshare database with the following link: https://doi.org/10.6084/m9.figshare.28599722

## References

[1] J. I. Gray , D. L. Farber , Annu. Rev. Immunol. 2022, 40, 195. 35044795 10.1146/annurev-immunol-093019-112809 PMC11194112

[2] L. C. Davies , S. J. Jenkins , J. E. Allen , P. R. Taylor , Nat. Immunol. 2013, 14, 986. 24048120 10.1038/ni.2705 PMC4045180

[3] M. A. Wallace , Aorn j 1998, 68, 799.

[4] C. Hinze , N. Karaiskos , A. Boltengagen , K. Walentin , K. Redo , N. Himmerkus , M. Bleich , S. S. Potter , A. S. Potter , K. U. Eckardt , C. Kocks , N. Rajewsky , K. M. Schmidt‐Ott , J. Am. Soc. Nephrol. 2021, 32, 291. 33239393 10.1681/ASN.2020070930 PMC8054904

[5] B. J. Stewart , J. R. Ferdinand , M. D. Young , T. J. Mitchell , K. W. Loudon , A. M. Riding , N. Richoz , G. L. Frazer , J. U. L. Staniforth , F. A. Vieira Braga , R. A. Botting , D. M. Popescu , R. Vento‐Tormo , E. Stephenson , A. Cagan , S. J. Farndon , K. Polanski , M. Efremova , K. Green , M. Del Castillo Velasco‐Herrera , C. Guzzo , G. Collord , L. Mamanova , T. Aho , J. N. Armitage , A. C. P. Riddick , I. Mushtaq , S. Farrell , D. Rampling , J. Nicholson , et al., Science 2019, 365, 1461. 31604275 10.1126/science.aat5031 PMC7343525

[6] A. M. C. Böhner , A. Effland , A. M. Jacob , K. A. M. Böhner , Z. Abdullah , S. Brähler , U. I. Attenberger , M. Rumpf , C. Kurts , Kidney Int. 2024, 105, 1254. 38458475 10.1016/j.kint.2024.01.043

[7] M. Brezis , S. N. Heyman , D. Dinour , F. H. Epstein , S. Rosen , J. Clin. Invest. 1991, 88, 390. 1864953 10.1172/JCI115316 PMC295343

[8] C. J. Lee , B. S. Gardiner , R. G. Evans , D. W. Smith , Am. J. Physiol. Renal. Physiol. 2018, 315, F1787. 30256129 10.1152/ajprenal.00363.2018

[9] R. G. Evans , D. W. Smith , C.‐J. Lee , J. P. Ngo , B. S. Gardiner , The Anatom. Rec. 2020, 303, 2544. 10.1002/ar.24260 31566903

[10] A. Goldspink , J. Schmitz , O. Babyak , N. Brauns , J. Milleck , A. M. Breloh , S. V. Fleig , K. Jobin , L. Schwarz , H. Haller , F. Wagenlehner , J. H. Bräsen , C. Kurts , S. von Vietinghoff , Kidney Int. 2023, 104, 279. 37098380 10.1016/j.kint.2023.03.034

[11] C. Kurts , F. Ginhoux , U. Panzer , Nat. Rev. Nephrol. 2020, 16, 391. 32372062 10.1038/s41581-020-0272-y

[12] C. Kurts , S. von Vietinghoff , C. F. Krebs , U. Panzer , Nat. Rev. Immunol. 2025, 25, 460. 39885266 10.1038/s41577-025-01131-y

[13] V. Marx , Nat. Methods 2021, 18, 9. 34413524 10.1038/s41592-021-01258-5

[14] A. Rao , D. Barkley , G. S. França , I. Yanai , Nature 2021, 596, 211. 34381231 10.1038/s41586-021-03634-9 PMC8475179

[15] L. Moses , L. Pachter , Nat. Methods 2022, 19, 534. 35273392 10.1038/s41592-022-01409-2

[16] J. Engesser , R. Khatri , D. P. Schaub , Y. Zhao , H. J. Paust , Z. Sultana , N. Asada , J. H. Riedel , V. Sivayoganathan , A. Peters , A. Kaffke , S. L. Jauch‐Speer , T. Goldbeck‐Strieder , V. G. Puelles , U. O. Wenzel , O. M. Steinmetz , E. Hoxha , J. E. Turner , H. W. Mittrücker , T. Wiech , T. B. Huber , S. Bonn , C. F. Krebs , U. Panzer , Nat. Commun. 2024, 15, 8220. 39300109 10.1038/s41467-024-52525-w PMC11413367

[17] D. Ramsköld , G.‐J. Hendriks , A. J. M. Larsson , J. V. Mayr , C. Ziegenhain , M. Hagemann‐Jensen , L. Hartmanis , R. Sandberg , Nat. Cell Biol. 2024, 26, 1725. 39198695 10.1038/s41556-024-01486-9 PMC11469958

[18] W. Jin , J. Pei , J. R. Roy , S. Jayaraman , R. M. Ahalliya , G. V. Kanniappan , M. Mironescu , C. P. Palanisamy , Ageing Res. Rev. 2024, 100, 102454. 39142391 10.1016/j.arr.2024.102454

[19] J. Mao , H. You , M. Wang , Y. Ba , J. Qian , P. Cheng , C. Lu , J. Chen , Kidney Int. 2024, 105, 562. 38142040 10.1016/j.kint.2023.11.027

[20] B. Yoon , H. Kim , S. W. Jung , J. Park , Kidney Int. 2024, 105, 1186. 38554991 10.1016/j.kint.2024.01.045

[21] N. Peiffer‐Smadja , T. M. Rawson , R. Ahmad , A. Buchard , P. Georgiou , F. X. Lescure , G. Birgand , A. H. Holmes , Clin Microbiol. Infect. 2020, 26, 584. 31539636 10.1016/j.cmi.2019.09.009

[22] Q. Ma , D. Xu , Nat. Rev. Mol. Cell Biol. 2022, 23, 303. 35197610 10.1038/s41580-022-00466-x PMC8864973

[23] D. Molho , J. Ding , W. Tang , Z. Li , H. Wen , Y. Wang , J. Venegas , W. Jin , R. Liu , R. Su , P. Danaher , R. Yang , Y. L. Lei , Y. Xie , J. Tang , ACM Trans. Intell. Syst. Technol. 2024, 15, 1.

[24] K. Asada , K. Takasawa , H. Machino , S. Takahashi , N. Shinkai , A. Bolatkan , K. Kobayashi , M. Komatsu , S. Kaneko , K. Okamoto , R. Hamamoto , Biomedicines 2021, 9, 1513. 34829742 10.3390/biomedicines9111513 PMC8614827

[25] S. A. Dick , A. Wong , H. Hamidzada , S. Nejat , R. Nechanitzky , S. Vohra , B. Mueller , R. Zaman , C. Kantores , L. Aronoff , A. Momen , D. Nechanitzky , W. Y. Li , P. Ramachandran , S. Q. Crome , B. Becher , M. I. Cybulsky , F. Billia , S. Keshavjee , S. Mital , C. S. Robbins , T. W. Mak , S. Epelman , Sci Immunol 2022, 7, abf7777. 10.1126/sciimmunol.abf7777 34995099

[26] C. K. Weisheit , D. R. Engel , C. Kurts , Clin. J. Am. Soc. Nephrol. 2015, 10, 1841. 25568218 10.2215/CJN.07100714 PMC4594071

[27] N. Yin , W. Zhang , X. X. Sun , R. Wei , Q. Yang , F. He , C. Li , L. Guo , M. Feng , Cell Rep. Med. 2023, 4, 101132. 37541252 10.1016/j.xcrm.2023.101132 PMC10439255

[28] C. K. Weisheit , D. R. Engel , C. Kurts , Clin. J. Am. Soc. Nephrol. 2015, 10, 1841. 25568218 10.2215/CJN.07100714 PMC4594071

[29] J. Yin , Q. Mei , M. Prinz , Z. Abdullah , U. Panzer , J. Li , S. von Vietinghoff , C. Kurts , Kidney Int. 2023, 104, 605. 37290602 10.1016/j.kint.2023.04.031

[30] A. Ransick , N. O. Lindstrom , J. Liu , Q. Zhu , J. J. Guo , G. F. Alvarado , A. D. Kim , H. G. Black , J. Kim , A. P. McMahon , Dev. Cell 2019, 51, 399. 31689386 10.1016/j.devcel.2019.10.005 PMC6948019

[31] V. H. Haase , Kidney Int. 2015, 88, 213. 26230196 10.1038/ki.2015.89 PMC4534718

[32] F. Liu , S. Dai , D. Feng , Z. Qin , X. Peng , S. Sakamuri , M. Ren , L. Huang , M. Cheng , K. E. Mohammad , P. Qu , Y. Chen , C. Zhao , F. Zhu , S. Liang , B. H. Aktas , X. Yang , H. Wang , P. V. G. Katakam , D. W. Busija , T. Fischer , P. K. Datta , J. Rappaport , B. Gao , X. Qin , Nat. Commun. 2020, 11, 2280. 32385245 10.1038/s41467-020-16158-z PMC7210253

[33] S. Ide , Y. Yahara , Y. Kobayashi , S. A. Strausser , K. Ide , A. Watwe , S. Xu‐Vanpala , J. R. Privratsky , S. D. Crowley , M. L. Shinohara , B. A. Alman , T. Souma , Elife 2020, 9, 1756. 10.7554/eLife.51756 PMC7205460 32301704

[34] S. A. Dick , A. Wong , H. Hamidzada , S. Nejat , R. Nechanitzky , S. Vohra , B. Mueller , R. Zaman , C. Kantores , L. Aronoff , A. Momen , D. Nechanitzky , W. Y. Li , P. Ramachandran , S. Q. Crome , B. Becher , M. I. Cybulsky , F. Billia , S. Keshavjee , S. Mital , C. S. Robbins , T. W. Mak , S. Epelman , Sci. Immunol. 2022, 7, abf7777. 10.1126/sciimmunol.abf7777 34995099

[35] K. A. Zimmerman , M. R. Bentley , J. M. Lever , Z. Li , D. K. Crossman , C. J. Song , S. Liu , M. R. Crowley , J. F. George , M. Mrug , B. K. Yoder , J. Am. Soc. Nephrol. 2019, 30, 767. 30948627 10.1681/ASN.2018090931 PMC6493978

[36] A. Zeisel , H. Hochgerner , P. Lönnerberg , A. Johnsson , F. Memic , J. van der Zwan , M. Häring , E. Braun , L. E. Borm , G. L. Manno , S. Codeluppi , A. Furlan , K. Lee , N. Skene , K. D. Harris , J. Hjerling‐Leffler , E. Arenas , P. Ernfors , U. Marklund , S. Linnarsson , Cell 2018, 174, 999. 30096314 10.1016/j.cell.2018.06.021 PMC6086934

[37] Y.‐L. Tan , Y. Yuan , L. Tian , Mol. Psychiatry 2020, 25, 351. 31772305 10.1038/s41380-019-0609-8 PMC6974435

[38] A. Saunders , E. Z. Macosko , A. Wysoker , M. Goldman , F. M. Krienen , H. de Rivera , E. Bien , M. Baum , L. Bortolin , S. Wang , A. Goeva , J. Nemesh , N. Kamitaki , S. Brumbaugh , D. Kulp , S. A. McCarroll , Cell 2018, 174, 1015. 30096299 10.1016/j.cell.2018.07.028 PMC6447408

[39] Q. Cao , D. C. H. Harris , Y. Wang , Physiology 2015, 30, 183. 25933819 10.1152/physiol.00046.2014

[40] B. R. Conway , E. D. O'Sullivan , C. Cairns , J. O'Sullivan , D. J. Simpson , A. Salzano , K. Connor , P. Ding , D. Humphries , K. Stewart , O. Teenan , R. Pius , N. C. Henderson , C. Benezech , P. Ramachandran , D. Ferenbach , J. Hughes , T. Chandra , L. Denby , J. Am. Soc. Nephrol. 2020, 31, 2833. 32978267 10.1681/ASN.2020060806 PMC7790206

[41] K. Hochheiser , D. R. Engel , L. Hammerich , F. Heymann , P. A. Knolle , U. Panzer , C. Kurts , J. Am. Soc. Nephrol. 2011, 22, 306. 21164025 10.1681/ASN.2010050548 PMC3029903

[42] V. Mysore , S. Tahir , K. Furuhashi , J. Arora , F. Rosetti , X. Cullere , P. Yazbeck , M. Sekulic , M. E. Lemieux , S. Raychaudhuri , B. H. Horwitz , T. N. Mayadas , J. Exp. Med. 2022, 219, 0210562. 10.1084/jem.20210562 PMC9006314 35404389

[43] W. Yao , Y. Chen , Z. Li , J. Ji , A. You , S. Jin , Y. Ma , Y. Zhao , J. Wang , L. Qu , H. Wang , C. Xiang , S. Wang , G. Liu , F. Bai , L. Yang , Adv. Sci. (Weinh) 2022, 9, 2103675. 35112806 10.1002/advs.202103675 PMC9036000

[44] K. Miyake , J. Ito , K. Takahashi , J. Nakabayashi , F. Brombacher , S. Shichino , S. Yoshikawa , S. Miyake , H. Karasuyama , Nat. Commun. 2024, 15, 1666. 38396021 10.1038/s41467-024-46148-4 PMC10891131

[45] Z. Liu , H. Wang , Z. Li , R. J. Dress , Y. Zhu , S. Zhang , D. De Feo , W. T. Kong , P. Cai , A. Shin , C. Piot , J. Yu , Y. Gu , M. Zhang , C. Gao , L. Chen , H. Wang , M. Vétillard , P. Guermonprez , I. Kwok , L. G. Ng , S. Chakarov , A. Schlitzer , B. Becher , C. A. Dutertre , B. Su , F. Ginhoux , Immunity 2023, 56, 1761. 37506694 10.1016/j.immuni.2023.07.001

[46] D. Janosevic , J. Myslinski , T. W. McCarthy , A. Zollman , F. Syed , X. Xuei , H. Gao , Y. L. Liu , K. S. Collins , Y. H. Cheng , S. Winfree , T. M. El‐Achkar , B. Maier , R. M. Ferreira , M. T. Eadon , T. Hato , P. C. Dagher , Elife 2021, 10, 2270. 10.7554/eLife.62270 PMC7810465 33448928

[47] Y. Dai , P. Jia , Y. Fang , H. Liu , X. Jiao , J. C. He , X. Ding , Sci. Rep. 2016, 6, 27091. 27250735 10.1038/srep27091 PMC4890025

[48] R. Keepers Tiffany , K. Gross Lisa , G. Obrig Tom , Infect. Immun. 2007, 75, 1229. 17220320 10.1128/IAI.01663-06 PMC1828550

[49] J. R. Privratsky , S. Ide , Y. Chen , H. Kitai , J. Ren , H. Fradin , X. Lu , T. Souma , S. D. Crowley , Kidney Int. 2023, 103, 514. 36334787 10.1016/j.kint.2022.10.008 PMC9974788

[50] M. S. Balzer , T. Doke , Y. W. Yang , D. L. Aldridge , H. Hu , H. Mai , D. Mukhi , Z. Ma , R. Shrestha , M. B. Palmer , C. A. Hunter , K. Susztak , Nat. Commun. 2022, 13, 4018. 35821371 10.1038/s41467-022-31772-9 PMC9276703

[51] T. A. Schiffer , H. Gustafsson , F. Palm , Am. J. Physiol. Renal. Physiol. 2018, 315, F677. 29846107 10.1152/ajprenal.00207.2018

[52] Y.‐J. Day , L. Huang , H. Ye , J. Linden , M. D. Okusa , Am. J. Phys.‐Renal Phys. 2005, 288, F722. 10.1152/ajprenal.00378.2004 15561971

[53] H. Wu , R. G. Villalobos , X. Yao , D. Reilly , T. Chen , M. Rankin , E. Myshkin , M. D. Breyer , B. D. Humphreys , Cell Metab. 2022, 34, 1064. 35709763 10.1016/j.cmet.2022.05.010 PMC9262852

[54] H.‐D. Li , Y.‐K. You , B.‐Y. Shao , W.‐F. Wu , Y.‐F. Wang , J.‐B. Guo , X.‐M. Meng , H. Chen , Front. Immunol. 2022, 13, 2022. 10.3389/fimmu.2022.1015142 PMC9666695 36405700

[55] B. Zhang , Y. Wu , Z. Wang , S. Gao , H. Liu , Y. Lin , P. Yu , Front. Immunol. 2025, 16, 1521554. 40046045 10.3389/fimmu.2025.1521554 PMC11879818

[56] C. Q. F. Klessens , M. Zandbergen , R. Wolterbeek , J. A. Bruijn , T. J. Rabelink , I. M. Bajema , D. H. T. IJpelaar , Nephrol., Dial., Transplant. 2016, 32, 1322. 10.1093/ndt/gfw260 27416772

[57] V. Perkovic , M. J. Jardine , B. Neal , S. Bompoint , H. J. L. Heerspink , D. M. Charytan , R. Edwards , R. Agarwal , G. Bakris , S. Bull , C. P. Cannon , G. Capuano , P. L. Chu , D. de Zeeuw , T. Greene , A. Levin , C. Pollock , D. C. Wheeler , Y. Yavin , H. Zhang , B. Zinman , G. Meininger , B. M. Brenner , K. W. Mahaffey , N. Engl. J. Med. 2019, 380, 2295.30990260

[58] D. Z. I. Cherney , B. A. Perkins , N. Soleymanlou , M. Maione , V. Lai , A. Lee , N. M. Fagan , H. J. Woerle , O. E. Johansen , U. C. Broedl , M. von Eynatten , Circulation 2014, 129, 587. 24334175 10.1161/CIRCULATIONAHA.113.005081

[59] G. L. Bakris , M. Siomos , D. Richardson , I. Janssen , W. K. Bolton , L. Hebert , R. Agarwal , D. Catanzaro , Kidney Int. 2000, 58, 2084. 11044229 10.1111/j.1523-1755.2000.00381.x

[60] E. J. Lewis , L. G. Hunsicker , R. P. Bain , R. D. Rohde , N. Engl. J. Med. 1993, 329, 1456. 8413456 10.1056/NEJM199311113292004

[61] Y. Guan , C. Hao , D. R. Cha , R. Rao , W. Lu , D. E. Kohan , M. A. Magnuson , R. Redha , Y. Zhang , M. D. Breyer , Nat. Med. 2005, 11, 861. 16007095 10.1038/nm1278

[62] R. W. Nesto , D. Bell , R. O. Bonow , V. Fonseca , S. M. Grundy , E. S. Horton , M. L. Winter , D. Porte , C. F. Semenkovich , S. Smith , L. H. Young , R. Kahn , Diabet. Care 2004, 27, 256. 10.2337/diacare.27.1.256 14693998

[63] L. C. Davies , S. J. Jenkins , J. E. Allen , P. R. Taylor , Nat. Immunol. 2013, 14, 986. 24048120 10.1038/ni.2705 PMC4045180

[64] G. Caputa , A. Castoldi , E. J. Pearce , Nat. Immunol. 2019, 20, 793. 31213715 10.1038/s41590-019-0407-0

[65] J. W. Griffith , C. L. Sokol , A. D. Luster , Annu. Rev. Immunol. 2014, 32, 659. 24655300 10.1146/annurev-immunol-032713-120145

[66] A. A. Tamouridou , X. E. Pantazi , T. Alexandridis , A. Lagopodi , G. Kontouris , D. Moshou , Sensors 2018, 18, 2770. 30142904 10.3390/s18092770 PMC6163850

[67] N. Moriel , E. Senel , N. Friedman , N. Rajewsky , N. Karaiskos , M. Nitzan , Nat. Protoc. 2021, 16, 4177. 34349282 10.1038/s41596-021-00573-7

[68] T. Biancalani , G. Scalia , L. Buffoni , R. Avasthi , Z. Lu , A. Sanger , N. Tokcan , C. R. Vanderburg , Å. Segerstolpe , M. Zhang , I. Avraham‐Davidi , S. Vickovic , M. Nitzan , S. Ma , A. Subramanian , M. Lipinski , J. Buenrostro , N. B. Brown , D. Fanelli , X. Zhuang , E. Z. Macosko , A. Regev , Nat. Meth. 2021, 18, 1352. 10.1038/s41592-021-01264-7 PMC8566243 34711971

[69] R. Wei , S. He , S. Bai , E. Sei , M. Hu , A. Thompson , K. Chen , S. Krishnamurthy , N. E. Navin , Nat. Biotechnol. 2022, 40, 1190. 35314812 10.1038/s41587-022-01233-1 PMC9673606

[70] M. R. Vahid , E. L. Brown , C. B. Steen , W. Zhang , H. S. Jeon , M. Kang , A. J. Gentles , A. M. Newman , Nat. Biotechnol. 2023, 41, 1543. 36879008 10.1038/s41587-023-01697-9 PMC10635828

[71] J. Zhou , A. Abedini , M. S. Balzer , R. Shrestha , P. Dhillon , H. Liu , H. Hu , K. Susztak , J. Am. Soc. Nephrol. 2023, 34, 1843. 37639336 10.1681/ASN.0000000000000217 PMC10631616

[72] P. P. Kapitsinou , V. H. Haase , Am. J. Physiol.‐Renal Physiol. 2015, 309, F821. 26311114 10.1152/ajprenal.00224.2015 PMC4652073

[73] S. C. Huen , L. G. Cantley , Pediatr. Nephrol. 2015, 30, 199. 24442822 10.1007/s00467-013-2726-y PMC5048744

[74] H. Chen , N. Liu , S. Zhuang , Front. Immunol. 2022, 13, 934299. 35911736 10.3389/fimmu.2022.934299 PMC9326079

[75] O. Foresto‐Neto , A. da Silva , M. Cipelli , F. P. R. Santana‐Novelli , N. O. S. Camara , Kidney Res. Clin. Pract. 2023, 42, 561. 37448286 10.23876/j.krcp.23.012 PMC10565456

[76] N. Ma , H. Lu , N. Li , W. Ni , W. Zhang , Q. Liu , W. Wu , S. Xia , J. Wen , T. Zhang , Cell Death Dis. 2024, 15, 163. 38388468 10.1038/s41419-024-06525-9 PMC10883957

[77] H. J. L. Heerspink , B. V. Stefánsson , R. Correa‐Rotter , G. M. Chertow , T. Greene , F. F. Hou , J. F. E. Mann , J. J. V. McMurray , M. Lindberg , P. Rossing , C. D. Sjöström , R. D. Toto , A. M. Langkilde , D. C. Wheeler , N. Engl. J. Med. 2020, 383, 1436. 32970396 10.1056/NEJMoa2024816

[78] B. M. Brenner , M. E. Cooper , D. de Zeeuw , W. F. Keane , W. E. Mitch , H. H. Parving , G. Remuzzi , S. M. Snapinn , Z. Zhang , S. Shahinfar , N. Engl. J. Med. 2001, 345, 861. 11565518 10.1056/NEJMoa011161

[79] M. J. Hawrylycz , E. S. Lein , A. L. Guillozet‐Bongaarts , E. H. Shen , L. Ng , J. A. Miller , L. N. van de Lagemaat , K. A. Smith , A. Ebbert , Z. L. Riley , C. Abajian , C. F. Beckmann , A. Bernard , D. Bertagnolli , A. F. Boe , P. M. Cartagena , M. M. Chakravarty , M. Chapin , J. Chong , R. A. Dalley , B. D. Daly , C. Dang , S. Datta , N. Dee , T. A. Dolbeare , V. Faber , D. Feng , D. R. Fowler , J. Goldy , B. W. Gregor , et al., Nature 2012, 489, 391. 22996553 10.1038/nature11405 PMC4243026

[80] E. S. Lein , M. J. Hawrylycz , N. Ao , M. Ayres , A. Bensinger , A. Bernard , A. F. Boe , M. S. Boguski , K. S. Brockway , E. J. Byrnes , L. Chen , L. Chen , T. M. Chen , M. C. Chin , J. Chong , B. E. Crook , A. Czaplinska , C. N. Dang , S. Datta , N. R. Dee , A. L. Desaki , T. Desta , E. Diep , T. A. Dolbeare , M. J. Donelan , H. W. Dong , J. G. Dougherty , B. J. Duncan , A. J. Ebbert , G. Eichele , et al., Nature 2007, 445, 168. 17151600 10.1038/nature05453

[81] J. Yin , M. Eichler , D. P. Schaub , N. Asada , J. Engesser , C. Lisowski , H. J. Paust , C. K. Weisheit , J. Li , D. Klaus , N. Garbi , S. von Vietinghoff , C. F. Krebs , U. Panzer , C. Kurts , Sci. Transl. Med. 2025, 17, adu0351. 10.1126/scitranslmed.adu0351 41091917

