# Supplementary material for: Predicting Macrophage Spatial Localization from Single‐Cell Transcriptomes to Uncover Disease Mechanisms
Source: Adv Sci (Weinh). 2026 Feb 28;13(21):e10924. doi: 10.1002/advs.202410924 (PMC13073240; doi:10.1002/advs.202410924)
Supplement: Supplementary file 1 — Supporting Information [file ADVS-13-e10924-s001.docx]

Supplementary data for

**Predicting Macrophage Spatial Localization from Single-Cell Transcriptomes to Uncover Disease Mechanisms**

Junping Yin^1*^, Qi Mei^2,3*^, Hans-Joachim Paust^4^, Ning Song^4,5^, Yu Zhao^4^, Daniela Klaus^1^, Melanie Eichler^1^, Yijun Hua^6,7^, Jie Qin^8,9^, Weiting Cheng^10^, Christina K. Weisheit^11^, Veronika Lukacs-Kornek^1^, Isis Ludwig-Portugall^1^, Sibylle von Vietinghoff^12^, Christian F. Krebs^4,13^, Johanna Klughammer^14^, Ulf Panzer^4,13^, Christian Kurts^1,15#^, Jian Li^1#^

*^1^Institute of Molecular Medicine and Experimental Immunology (IMMEI), University Hospital Bonn, Germany*

*^2^Department of Oncology，Tongji Hospital, Tongji Medical College, Huazhong University of Science and Technology, Wuhan, Hubei, People's Republic of China*

*^3^Cancer Center, Shanxi Bethune Hospital, Shanxi Academy of Medical Sciences, Tongji Shanxi Hospital, Third Hospital of Shanxi Medical University, Taiyuan, Shanxi, People's Republic of China*

*^4^III. Department of Medicine, University Medical Center Hamburg-Eppendorf, Hamburg, Germany*

*^5^Renal Division, Department of Medicine, Beijing Anzhen Hospital, Capital Medical University, Beijing, China*

*^6^Department of Nasopharyngeal Carcinoma, Guangdong Key Laboratory of Nasopharyngeal Carcinoma Diagnosis and Therapy, Sun Yat-sen University Cancer Center*

*^7^State Key Laboratory of Oncology in South China, Sun Yat-sen University Cancer Center*

*^8^Department of Obstetrics, Maternal and Child Health Hospital of Guangxi Zhuang Autonomous Region, Nanning, P. R. China*

*^9^Birth Defects Prevention and Control Institute, Maternal and Child Health Hospital of Guangxi Zhuang Autonomous Region, Nanning, P. R. China*

*^10^Cancer Center, Renmin Hospital of Wuhan University, Wuhan, Hubei, P.R. China*

^11^Department of Anesthesiology and Intensive Care Medicine, University Hospital Bonn, Germany

^12^Nephrology Section, Medical Clinic 1, University Hospital Bonn, Bonn, Germany

^13^Hamburg Center for Translational Immunology, University Medical Center Hamburg-Eppendorf, Hamburg, Germany

^14^Gene Center and Department of Biochemistry, Ludwig-Maximilians-Universität München, Munich, Germany

^15^Department of Microbiology and Immunology, Doherty Institute for Infection and Immunity, University of Melbourne, Victoria, Australia

*Joint first authors

#joint senior authors

send correspondence to jianli@uni-bonn.de, or to [ckurts@uni-bonn.de](mailto:ckurts@uni-bonn.de)


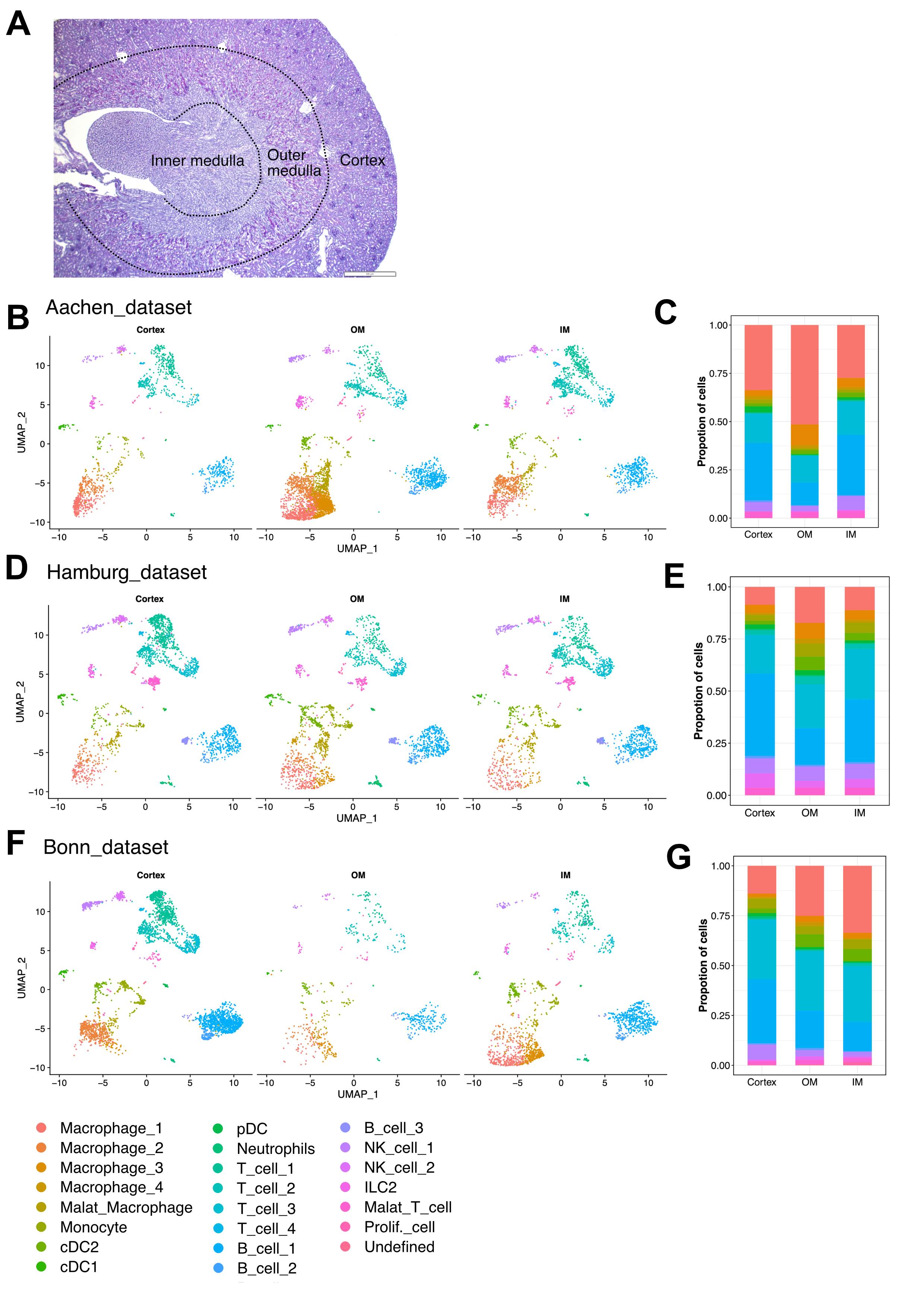


# Fig. S1. Single cell RNA sequencing datasets from Aachen, Hamburg and Bonn. A. Histological kidney section illustrating cortex, outer medulla, and inner medulla. B-F. UMAP visualization for the distribution of single cells from Aachen (B), Hamburg (D) and Bonn (F). Cell proportions for Aachen (C), Hamburg (E), and Bonn (G) datasets.


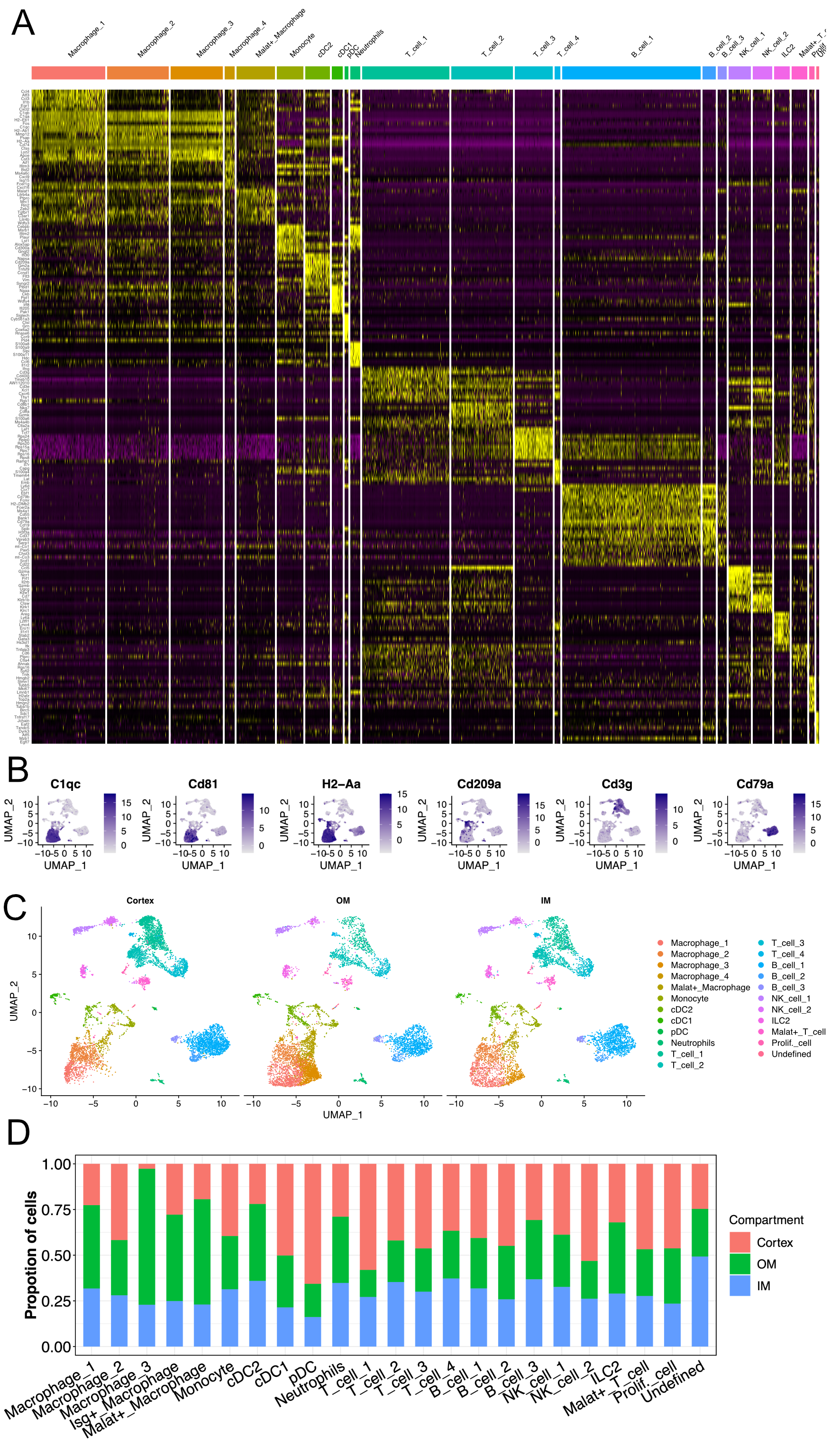


**Fig. S2. Single cell RNA sequencing of immune cells from the healthy kidney.** **A**. Heatmap showing the top 10 highest fold change differentially expressed markers of each cell subsets. **B**. Featureplots of markers for resident macrophages (C1qc, Cd81, H2-Aa), cDCs (Cd209a), T cells (Cd3g) and B cells (Cd79a). **C**. UMAP plot showing the immune cells in the three compartments of kidney. OM: outer medulla, IM: inner medulla. **D**. Proportions of renal immune cells in cortex, OM and IM in healthy mice.


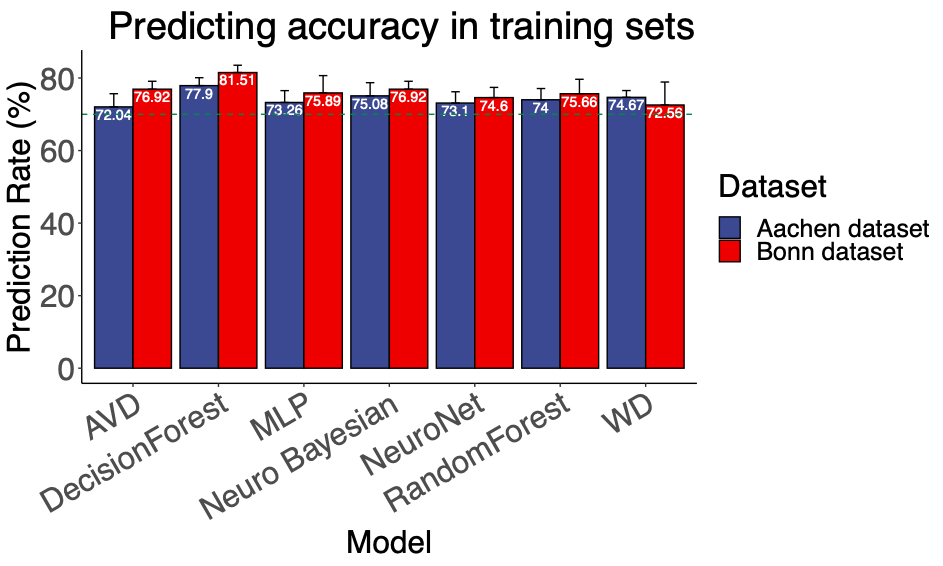


**Fig. S3.** **Predicting accuracy of the positioning of resident macrophages in the training datasets**: Aachen dataset (blue) and Bonn dataset (red). Green dashed line indicated the accuracy at 70%. Data was presented as Median ± SEM.


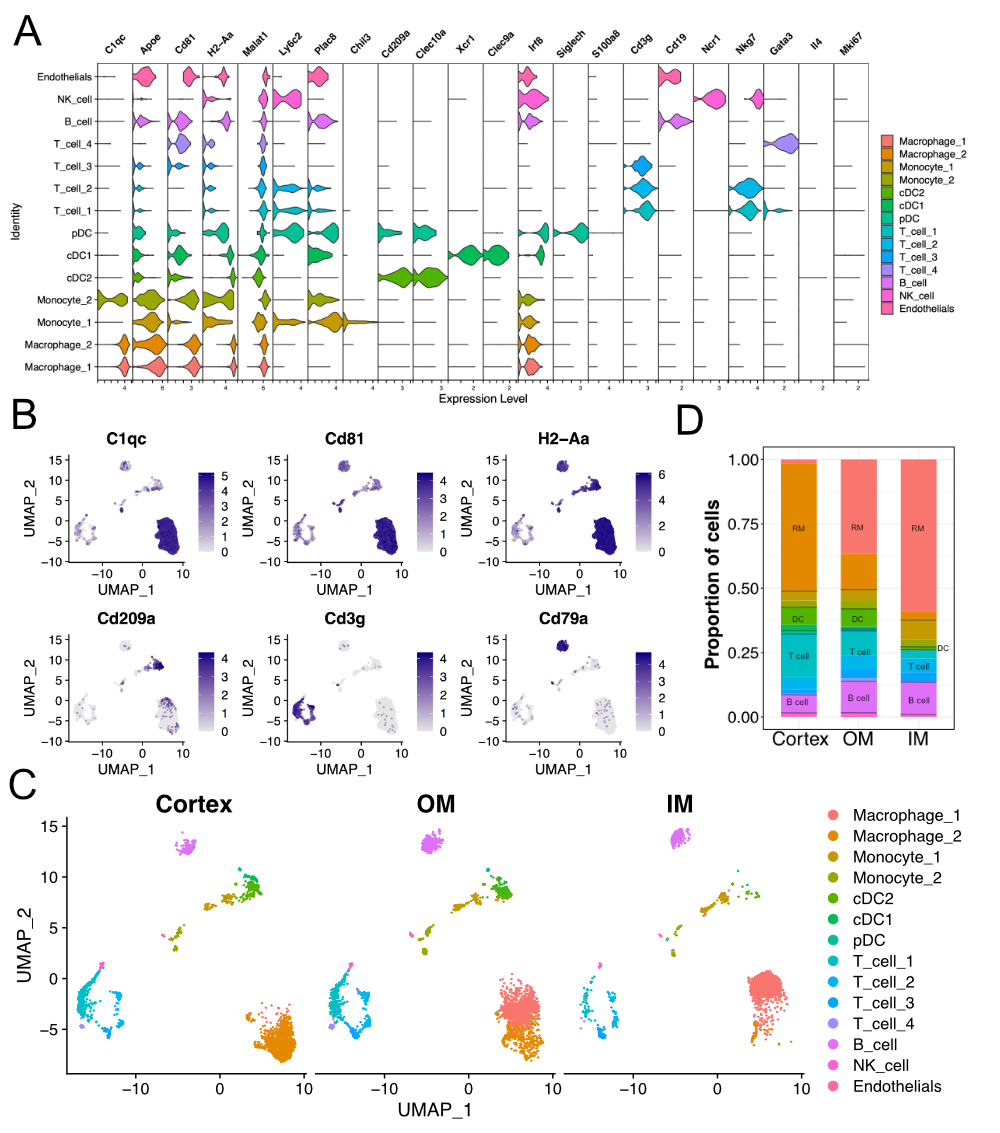


**Fig. S4.** **Single cell RNA sequencing of renal immune cells from Public dataset.** A. Violin plot showing expression of the markers used for annotation of immune cell subsets in the published dataset. B. Feature plot showing gene expression of the phenotype markers of resident macrophages (C1qc, Cd81, H2-Aa), cDC (H2-Aa, Cd209a), T cell (Cd3g) and B cell (Cd79a). C. UMAP plot showing immune cells in the three compartments of kidney in the public dataset. D. Cellular proportion of cell subsets of renal immune cell in cortex, OM and IM of the kidney.


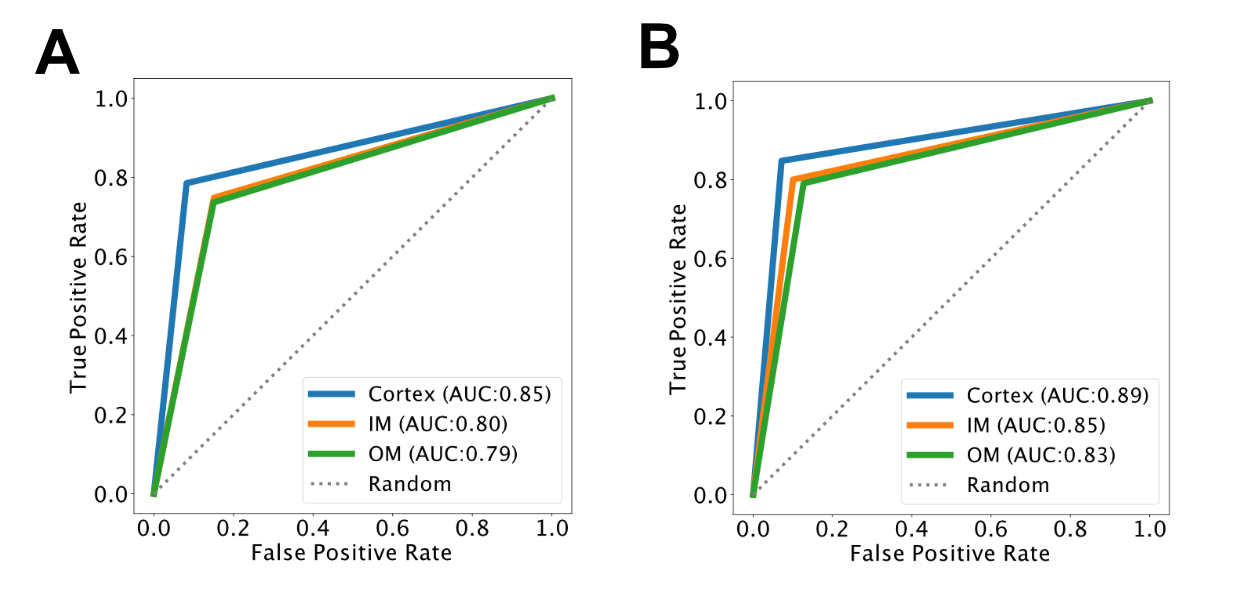

**Fig. S5. ROC curves illustrating external validation of the HVG251 gene set** for predicting the spatial positioning of resident macrophages in the Hamburg dataset (A) and the Public dataset (B). Panels (C) and (D) show the corresponding ROC curves using a randomized gene set for the same prediction task in the Hamburg dataset and the Public dataset, respectively. Panels (E) and (F) show the corresponding ROC curves using RFE251 gene set for the same prediction task in both validation datasets. Panels (G) and (H) show the corresponding ROC curves using MIR251 gene set for the same prediction task in both validation datasets. RFE: recursive feature elimination; MIR: mutual information-based ranking

**Fig. S6.** **GO enrichment analysis of shared genes between HVG251 and FAM104 geneset.** Bar plots showed the top 10 significantly enriched GO pathways of the shared genes between HVG251 and FAM104 geneset.


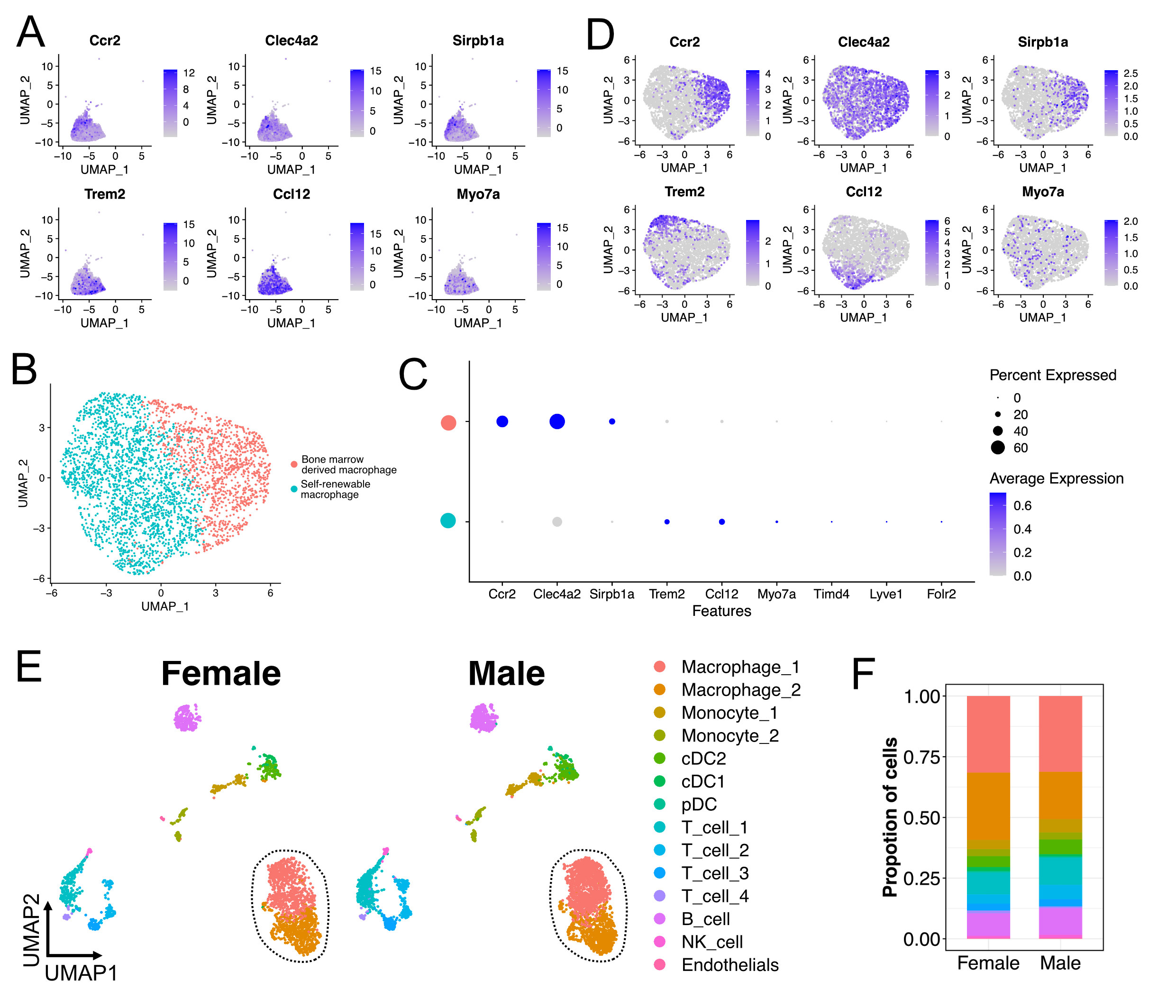


**Fig. S7. Lineage and sexual differences of resident macrophages in kidney of mouse.** **A**. Feature plots of markers for bone marrow-derived (Ccr2, Clec4a2, Sirpb1a) or self-renewable resident macrophages (Trem2, Ccl12, Myo7a) in Hamburg dataset. **B, C**. UMAP plot (B) and Dotplots (C) showing bone marrow-derived or self-renewable resident macrophages in the Hamburg dataset. **D**. Featureplots of markers for bone marrow-derived or self-renewable resident macrophages in the Public dataset. **E**. UMAP plot showing renal immune cells from male and female mice in the Public dataset. **F**. Cell proportions of immune cell subsets in female and male mice

.


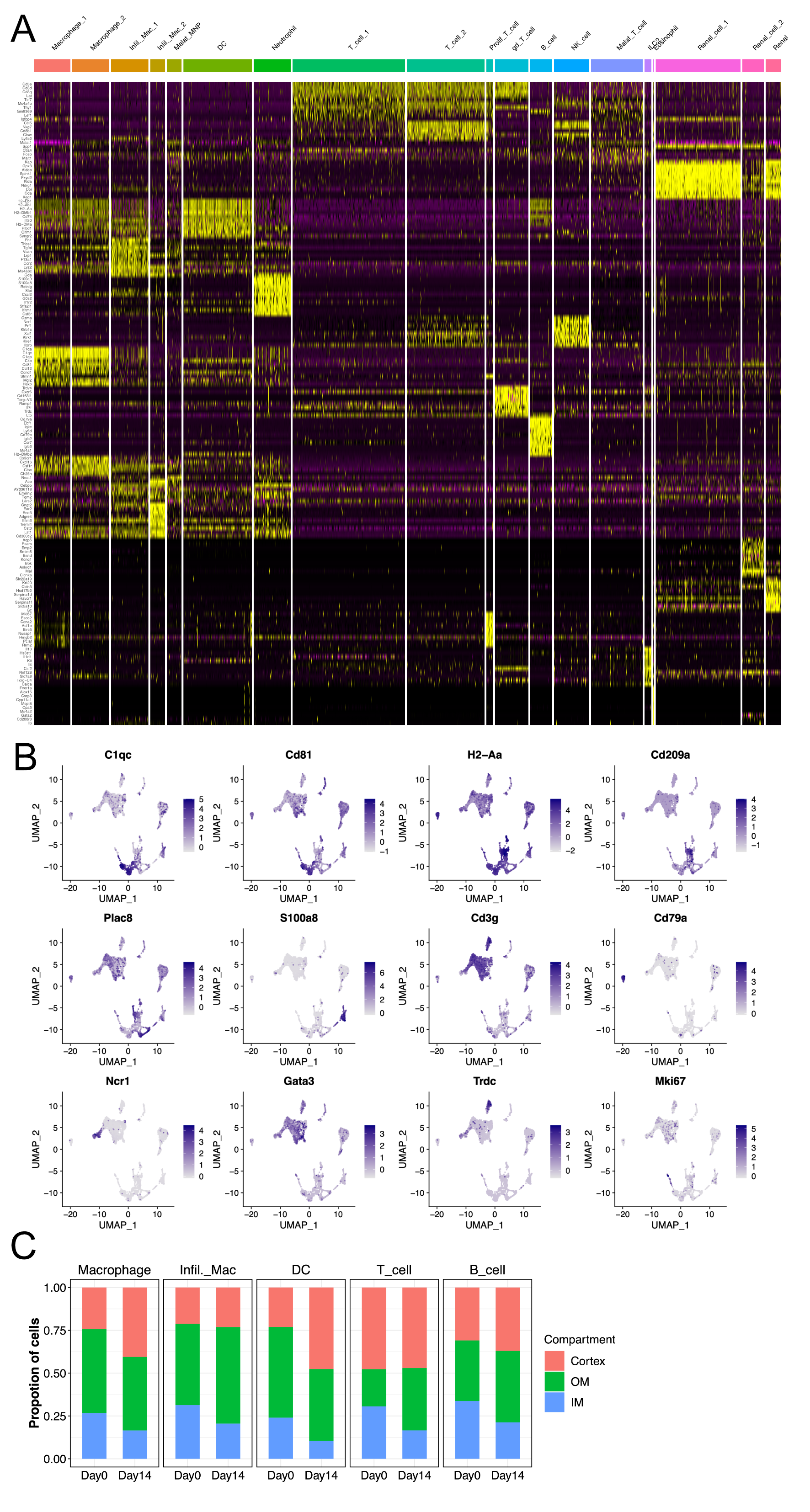


**Fig. S8. Single cell RNA sequencing of renal immune cells from cGN mice.** **A**. Heatmap showed the top 10 highest fold change differentially expressed markers of each cell subsets. **B**. Featureplots of markers for resident macrophages (C1qc, Cd81, H2-Aa), cDCs (Cd209a), infiltrated macrophages (Plac8), Neutrophils (S100a8), T cells (Cd3g), B cells (Cd79a), NK cells (Ncr1), ILC2 (Gata3), gdT cells (Trdc) and proliferating cells (Mki67). **C**. Distribution of Macrophages, Infiltrating macrophages, DCs, T cells and B cells over cortex, OM and IM in healthy kidney and cGN kidney.

**Fig. S9.** **Spatial prediction of the location of renal resident macrophages from mice with cGN.** ROC curve plots of prediction of positioning of resident macrophages at day 14 of cGN.


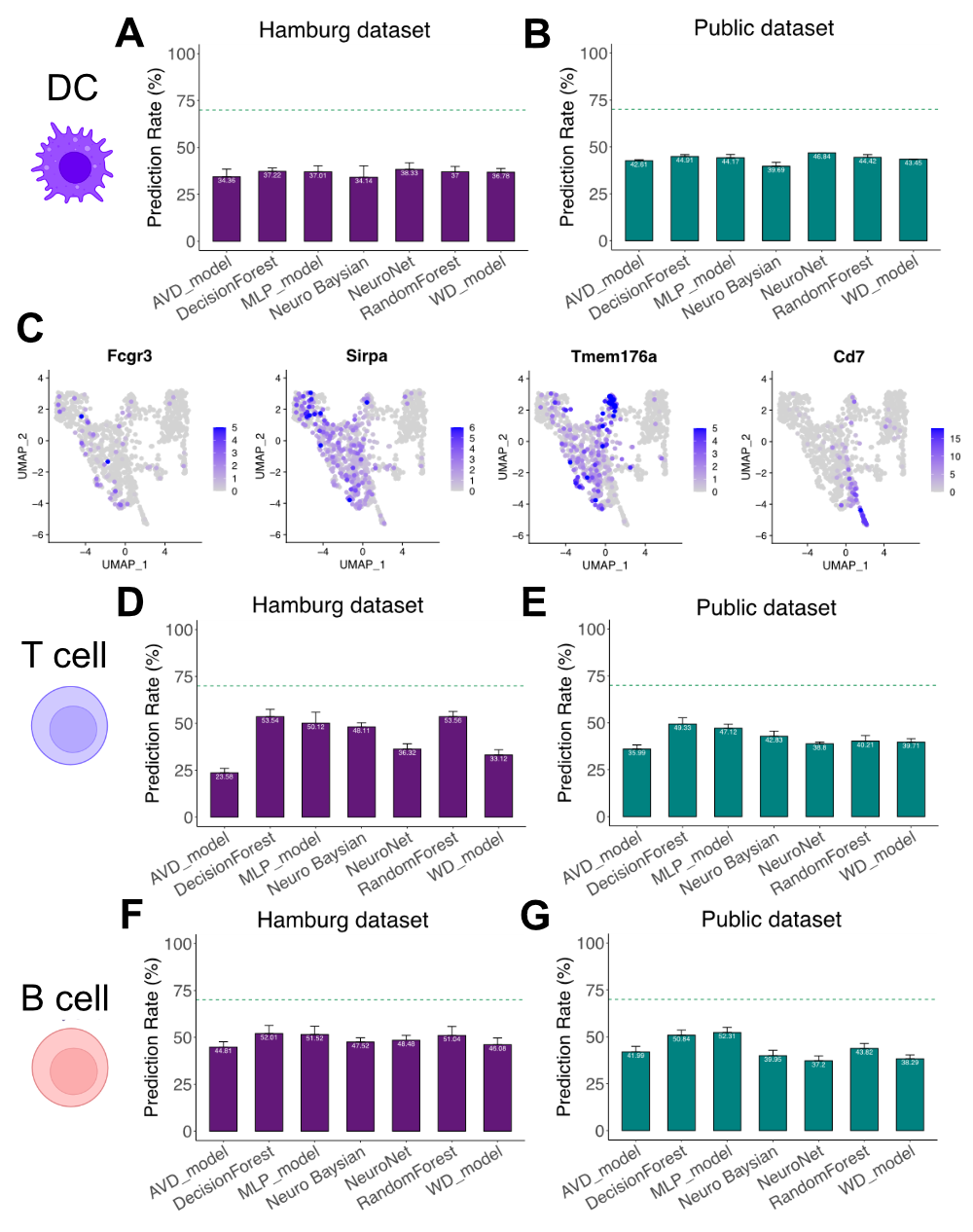


**Fig. S10.** **External validation for prediction of HVG251 and FAM104 geneset** in cDCs (A, B), T cells (D, E) and B cells (F, G) from the Hamburg (A, D, F) and Public dataset (B, E, G). Data presented as Median ± SEM. C. Feature plots showing gene expression levels of cellular phenotype markers (*Fcgr3*, *Sirpa*, *Tmem176a*, *Cd7*) in renal cDC.

**Fig. S11.** **Spatial prediction of kidney macrophage distribution across 9 different treatment conditions** 4a. db/db+AAV+ACEi(2d), 4b. db/db+AAV+ACEi(2w), 5a. db/db+AAV+Rosi(2d), 5b. db/db+AAV+Rosi(2w), 6a. db/db+AAV+ACEi+Rosi(2d), 6b. db/db+AAV+ACEi+Rosi(2w), 7a. db/db+AAV+SGLT2i(2d), 7b.db/db+AAV+ SGLT2i(2w), 8a. db/db+AAV+ACEi+SGLT2i(2d), 8b. db/db+AAV+ACEi+SGLT2i(2w). AAV: Adeno-associated virus, in that study, the authors used ReninAAV to generate diabetic kidney disease mouse model. Y-axis indicates cell numbers.
